# Identification, Purification and Molecular Characterization of Chondrosin, a New Protein with Anti-tumoral Activity from the Marine Sponge *Chondrosia Reniformis* Nardo 1847

**DOI:** 10.3390/md18080409

**Published:** 2020-08-02

**Authors:** Sonia Scarfì, Marina Pozzolini, Caterina Oliveri, Serena Mirata, Annalisa Salis, Gianluca Damonte, Daniela Fenoglio, Tiziana Altosole, Micha Ilan, Marco Bertolino, Marco Giovine

**Affiliations:** 1 Department of Earth, Environment and Life Sciences (DISTAV), University of Genova, Via Pastore 3, 16132 Genova, Italy; soniascarfi@unige.it (S.S.); marina.pozzolini@unige.it (M.P.); Caterina.oliveri@unige.it (C.O.); serena.mirata@edu.unige.it (S.M.); marco.bertolino@unige.it (M.B.); 2 Centro 3R, Interuniversitary Center for the Promotion of the Principles of the 3Rs in Teaching and Research, Via Caruso 16, 56122 Pisa, Italy; 3 Department of Experimental Medicine (DIMES), Biochemistry Section, University of Genova, Viale Benedetto XV 1, 16132 Genova, Italy; annalisa.salis@unige.it (A.S.); gianluca.damonte@unige.it (G.D.); Daniela.fenoglio@unige.it (D.F.); tiziana.alto@gmail.com (T.A.); 4 Centre of Excellence for Biomedical Research (CEBR), University of Genova, Viale Benedetto XV 9, 16132 Genova, Italy; 5 School of Zoology, Tel Aviv University, Tel Aviv 69978, Israel; milan@tauex.tau.ac.il

**Keywords:** Chondrosin, *Chondrosia reniformis*, marine toxin, cytotoxic protein, Porifera

## Abstract

*Chondrosia reniformis* is a common marine demosponge showing many peculiarities, lacking silica spicules and with a body entirely formed by a dense collagenous matrix. In this paper, we have described the identification of a new cytotoxic protein (chondrosin) with selective activity against specific tumor cell lines, from *C. reniformis*, collected from the Liguria Sea. Chondrosin was extracted and purified using a salting out approach and molecular weight size exclusion chromatography. The cytotoxic fractions were then characterized by two-dimensional gel electrophoresis and mass spectrometry analysis and matched the results with *C. reniformis* transcriptome database. The procedure allowed for identifying a full-length cDNA encoding for a 199-amino acids (aa) polypeptide, with a signal peptide of 21 amino acids. The mature protein has a theoretical molecular weight of 19611.12 and an IP of 5.11. Cell toxicity assays showed a selective action against some tumor cell lines (RAW 264.7 murine leukemia cells in particular). Cell death was determined by extracellular calcium intake, followed by cytoplasmic reactive oxygen species overproduction. The in silico modelling of chondrosin showed a high structural homology with the *N*-terminal region of the ryanodine receptor/channel and a short identity with defensin. The results are discussed suggesting a possible specific interaction of chondrosin with the Cav 1.3 ion voltage calcium channel expressed on the target cell membranes.

## 1. Introduction

The marine sponge *Chondrosia reniformis* Nardo 1847 is a common marine demosponge, widely distributed along all the Mediterranean Sea and East Atlantic Ocean, where it inhabits both shallow and deep-water environments [1]. This demosponge lacks siliceous spicules and its body is entirely formed by a dense collagenous matrix with peculiar molecular features [2,3,4] with finely-regulated production [5]. This sponge is also characterized by good regenerative properties [6] whose molecular mechanisms have recently been described [7]. *C. reniformis* collagens pose remarkable biotechnology potential: their successful use has been demonstrated in cosmetic preparations [8], in drug delivery [9,10], in biomembrane production [11] and in the production of active peptides with biomedical targets [12]. The well-known industrial value of this marine resource has pushed scientists to find sustainable methods of exploitation, whether through the production of sponge collagen by recombinant approaches [13,14] or by aquaculture attempts [15]. In particular, the current attempts at *C. reniformis* farming are promising, even if not yet optimised for a sustainable production of sponge biomass on a large scale, but in the next future relevant improvements in this field are expected. Some sporadic observations on this peculiar animal also document the release of unknown toxic compound(s) in the water hosting the specimens after collection, able to kill other animals in the same tank and to cause skin irritation in humans. On the other hand, it is also known that *C. reniformis* in some areas of the Adriatic Sea was usually eaten, after cooking, possibly implying a thermolability of this toxic compound(s) [16].

The aim of this study was to fill in the gap of knowledge on the toxic compounds produced by this common sponge, and to investigate their possible applicability in biomedicine, specifically in the field of anticancer therapy. Based on the above-mentioned evidences, a chemical purification procedure from a crude extract of *C. reniformis* specimens, collected in the Ligurian Sea was performed, and a cell cytotoxicity assay was set up to verify its activity and its possible anti-tumour effect on different cancer cell lines. Various experimental strategies were used to assess the compounds chemical nature, and to define the range of molecular weight (MW) of these toxic component(s). Chromatographic fractionation of the crude extract, mass spectrometry (MS) analysis and *C. reniformis* transcriptome data mining, were then performed to identify the active compound and its three-dimensional (3D) structure. Finally, the mechanism of toxicity on four cancer cell lines, representatives of different typologies of tumours, was also investigated, to define its mode of action that causes cell death preferentially in cancer cells.

## 2. Results and Discussion

### 2.1. The Cytotoxicity of a Crude Extract (CE) of C. reniformis is Due to a Protein Fraction

**Figure 1:**
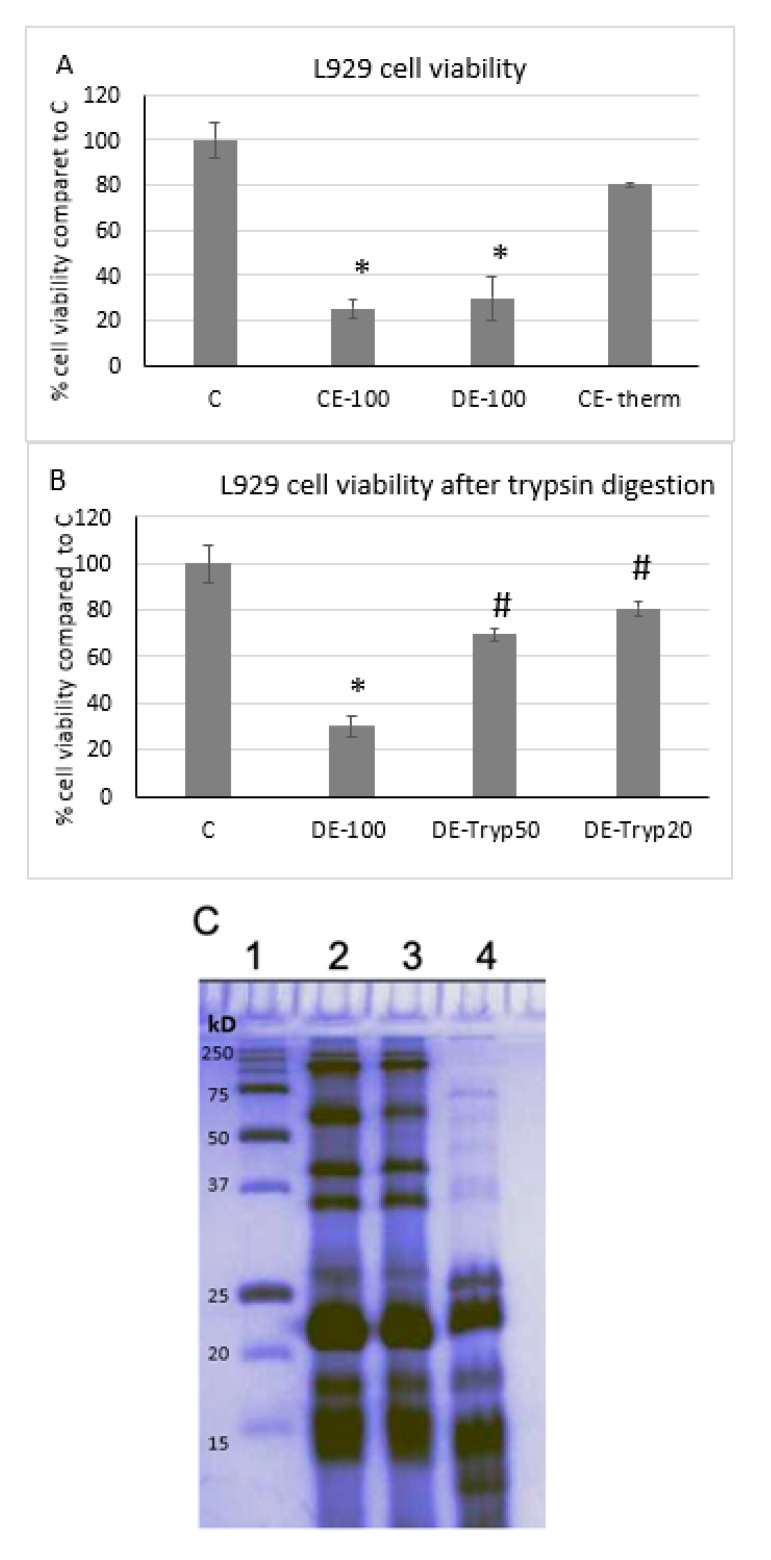
Cell toxicity evaluation. (**A**) L929 fibroblast cell growth quantitative evaluation, by the cell viability MTT test at 24 h, in the presence or absence of a 100-fold diluted crude extract (CE-100), of a 100-fold diluted dialysed extract (DE-100) and of a thermally-inactivated 100-fold diluted crude extract (CE-therm). Results are expressed as cell percentages relative to control cells (**C**) and are the mean ± Standard deviation (S.D). of three experiments performed in quadruplicate. Asterisks indicate significance in paired Tukey test (Analysis of Variance-ANOVA, *p* < 0.000001; Tukey vs. C: * *p* < 0.0001). (**B**) L929 fibroblast cell growth quantitative evaluation, by the cell viability MTT test at 24 h, in the presence or absence of a 100-fold diluted DE (DE-100) or of the same DE previously digested with two different concentrations of trypsin (DE-Tryp50 and DE-Tryp20, respectively). Results are expressed as cell percentages relative to control cells (C) and are the mean ± S.D. of three experiments performed in quadruplicate. Asterisks indicate significance in paired Tukey test (ANOVA, *p* < 0.000001; Tukey vs. C: * *p* < 0.0001; Tukey vs. DE-100: # *p* < 0.0001). (**C**) Sodium Dodecyl Sulphate-PolyAcrylamide Gel Electrophoresis (SDS-PAGE) of 20 μg of undiluted DE fraction after trypsin digestion for 24 h. Lane 1 molecular weight standards, Lane 2 untreated DE, lane 3 DE digested with tryspin 1:50 (*w:w*), lane 4 DE digested with trypsin 1:20 (*w:w*).

Preliminary experiments were performed on the crude hydrophilic extract (CE) of *C. reniformis* obtained by squeezing sponge specimens, collected in the Ligurian Sea. As shown in Figure 1A the RE exhibited a significant cytotoxic activity on L929 murine fibroblast cell line, where a 100-fold dilution of the CE caused 74.9 ± 4.5% cell death at 24 h incubation, analysed by the 3-(4,5-dimethylthiazol-2-yl)-2,5-diphenyltetrazolium bromide dye test (MTT) (CE-100 bar, *p* < 0.0001 compared to C). To determine if the cytotoxic activity of the CE was due to a small-molecule metabolite or to a protein, three different approaches were performed: a thermal sensitivity experiment, a 10,000 kDa cut-off dialysis of the CE (DE), and a trypsin digestion. Indeed, the CE cytotoxic component was sensitive to thermal treatment, as shown in Figure 1A (CE-therm bar), being completely inactivated after 10 min incubation at 100 °C. Conversely, the DE showed that the cytotoxic component was totally retained in the 10 kDa cut-off fraction (Figure 1A, DE-100 bar, 70.1 ± 9.7% cytotoxic activity compared to C, *p* < 0.0001). Finally, the trypsin digestion demonstrated a high degree of fragmentation into smaller peptides in the DE as shown in the gel electrophoresis analysis (Figure 1C lanes 3 and 4 compared to control lane 2) and L929 cells challenged with the trypsin-digested DE (Figure 1B, DE-Tryp20 and DE-Tryp50 bars) for 24 h showed a significant loss of the cytotoxic effect after the protease treatment compared to the untreated DE (Figure 1B, 57% loss of cytotoxic activity for DE-Tryp20 and 71% for DE-Tryp50, respectively, compared to DE-100, *p* < 0.0001 for both bars). These results clearly demonstrated that a significant cytotoxic activity on a mammalian cell viability of the DE fraction was due to one or more proteins. The production of toxic proteins is well known in marine sponges [17,18]. For biomedical applications, smaller molecules demonstrate a better exploitability than the larger ones [19], due to their easiness for in vitro and in vivo biological and pharmacological assays, even if there is not a true “dimensional” cut-off in the evaluation criteria for the pharmaceutical applicability of a polypeptide. Bioactivity, specificity of action, and low immunogenicity are the main keys for the successful use of a bioactive protein in therapy. The pharmacological target also plays a role, and like toxins of other marine and terrestrial organisms, the biomolecule’s action could have different levels of specificity. Proteins and peptides with selective action against bacteria and viruses are topics of increasing interest, whereas the research for new proteins and peptides with anti-tumour activity is the most promising.

### 2.2. Identification and Characterization of Chondrosin

**Figure 2:**
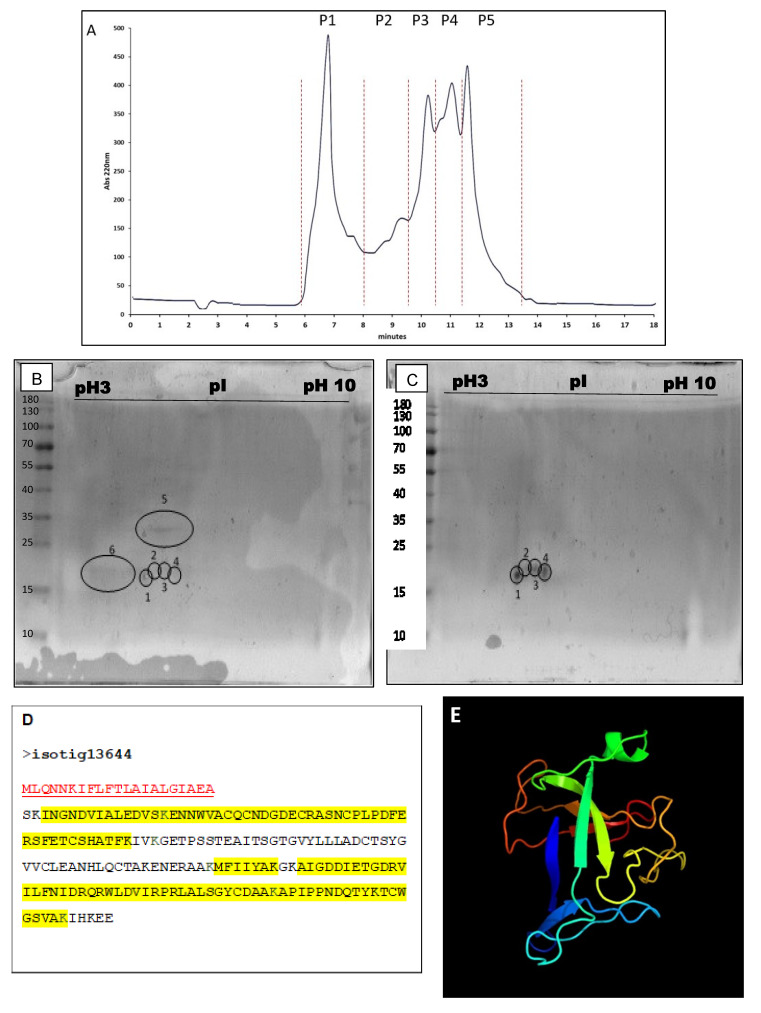
Identification by proteomic analysis. (**A**) Chromatogram at 220 nm absorbance obtained by HPLC (high-performance liquid chromatography) separation of the ammonium sulphate precipitate (ASP) fraction through a molecular weight size-exclusion column. The obtained peaks, from P1 to P5, were separately collected for further biological and chemical characterization. (**B**,**C**) Two-dimensional gel electrophoresis of *C. reniformis* HPLC purified P4 and P5 peaks, respectively. Samples were loaded in precast, 7-cm long, IPG strips with isoelectric focusing (IEF) pH 3–10. Spot protein identification (from spots 1 to 6) relative to peak 4 (**B**) and (from spot 1 to 4) relative to peak 5 (**C**), by MS analysis, are reported, respectively, in Table 1 and Table 2. Spots (1–4) are present in the same position in both gels corresponding to the same protein. (**D**) Chondrosin protein primary amino acid sequence. The identified signal peptide is in red, while the protein peptide coverage identified by MS analysis is highlighted in yellow. (**E**) Chondrosin predicted 3D structure using the Phyre^2^ web portal for protein modelling, prediction and analysis (http://www.sbg.bio.ic.ac.uk/phyre2/html/page.cgi?id=index © Structural Bioinformatics Group, Imperial College, London)

**Scheme 1:**
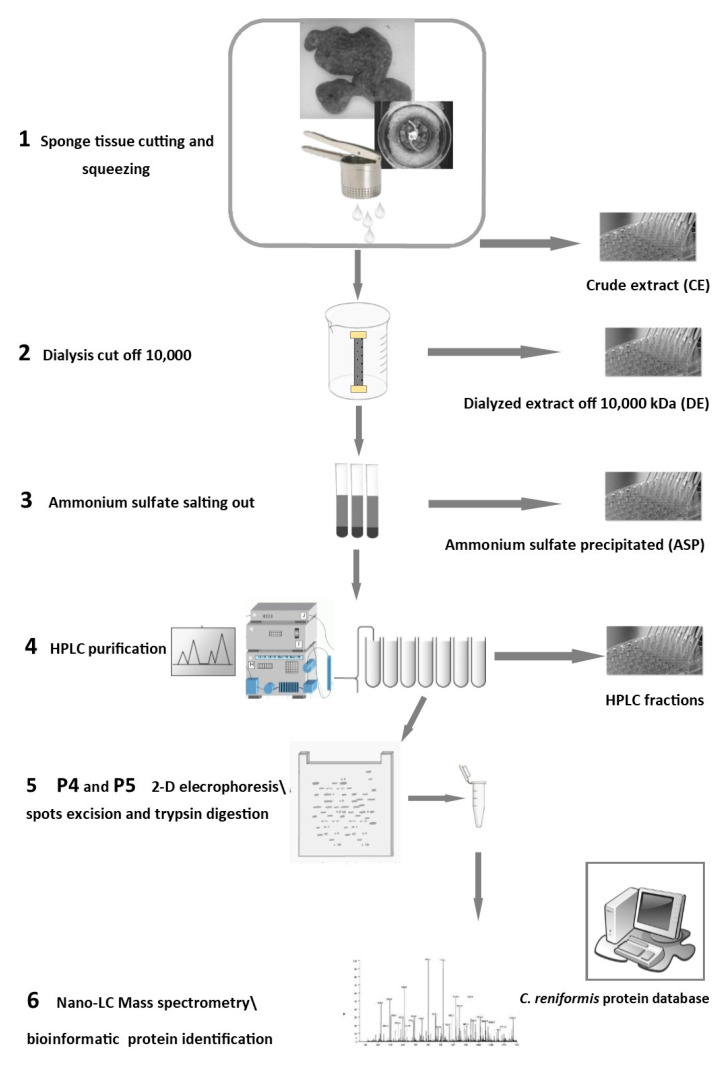
Procedure flowchart for the step-by-step purification and characterization of the cytotoxic/anti-tumour protein chondrosin from the marine sponge *C. reniformis.*

**Figure 3:**
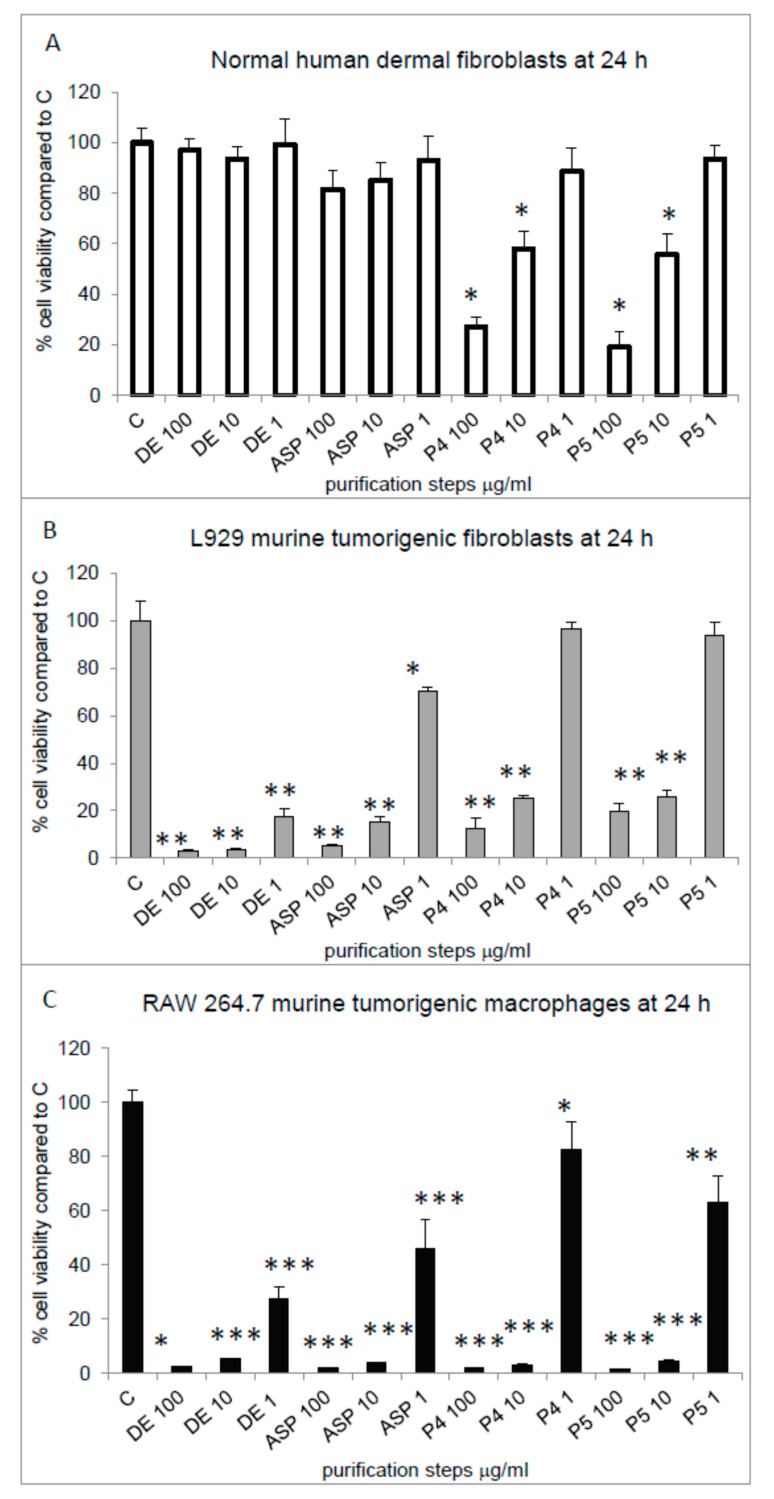

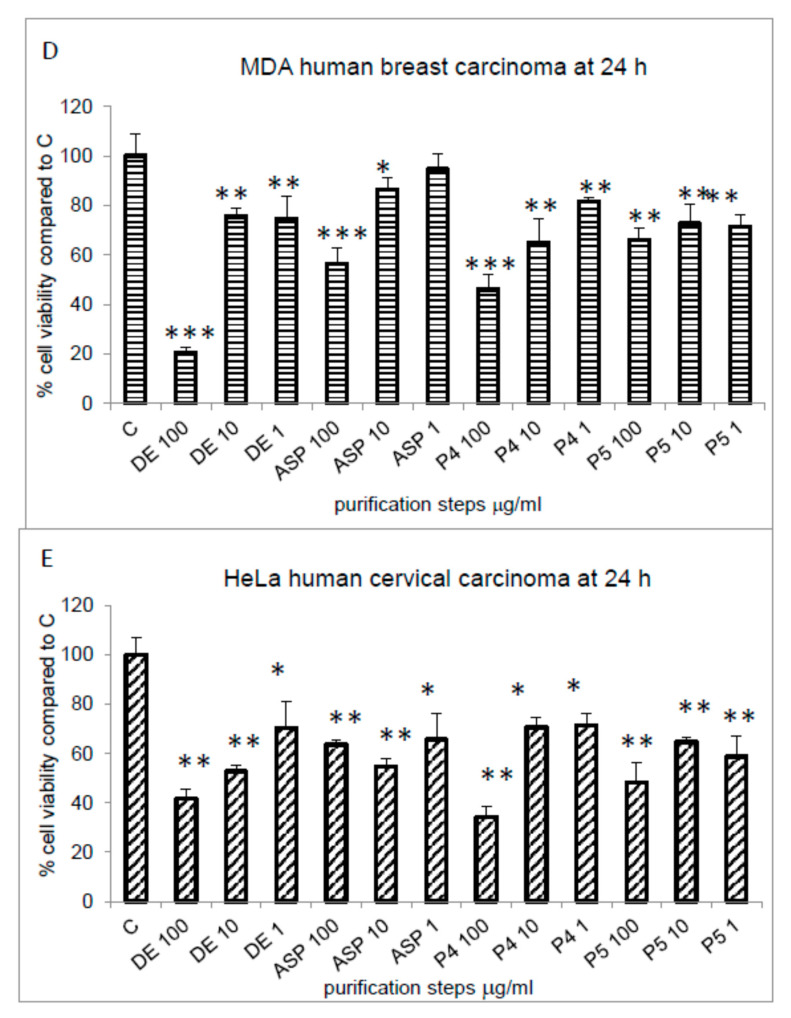
Cytotoxic/anti-tumour activity evaluation. (**A**) Normal human dermal fibroblast cell growth quantitative evaluation, by the cell viability MTT test at 24 h, in the presence or absence of various dilutions of *C. reniformis* purified fractions: dialysed extract (DE) from 1 to 100 μg/mL, ammonium sulfate precipitate extract (ASP) from 1 to 100 μg/mL, HPLC purified peak 4 (P4) and peak 5 (P5) from 1 to 100 μg/mL. Results are expressed as living cell percentages with respect to control cells (C) and are the mean ± S.D. of three experiments performed in quadruplicate. Asterisks indicate significance in paired Tukey test (ANOVA, *p* < 0.000001; Tukey vs. C: * *p* < 0.0001). (**B**) L929 murine tumorigenic fibroblasts cell growth quantitative evaluation in the same conditions as (A). Asterisks indicate significance in paired Tukey test (ANOVA, *p* < 0.000001; Tukey vs. C: ** *p* < 0.0001, * *p* < 0.01, respectively). (**C**) RAW 264.7 murine leukemia cell growth quantitative evaluation in the same conditions as (**A**). Asterisks indicate significance in paired Tukey test (ANOVA, *p* < 0.000001; Tukey vs. C: *** *p* < 0.0001, ** *p* < 0.0005, * *p* < 0.05, respectively). (**D**) MDA-MB-468 human breast carcinoma cell growth quantitative evaluation in the same conditions as (A). Asterisks indicate significance in paired Tukey test (ANOVA, *p* < 0.000001; Tukey vs. C: *** *p* < 0.0001, ** *p* < 0.005, * *p* < 0.05, respectively). (E) HeLa human cervical carcinoma cell growth quantitative evaluation in the same conditions as (A). Asterisks indicate significance in paired Tukey test (ANOVA, *p* < 0.000001; Tukey vs. C: ** *p* < 0.005, * *p* < 0.05, respectively).

In initial results (Figure 1), the main cytotoxic activity was confined to the protein fraction of the *C. reniformis* crude extract, therefore a biochemical protocol of protein fractionation was designed and tested to purify and identify the active protein(s) (Scheme 1). Thus, the RE was subjected to the following step-by-step purification procedure: (i) a 10 kDa-cut-off dialysis to get rid of small molecules and metabolites, and to obtain a dialysed extract fraction (DE); (ii) an ammonium sulphate (AS) biochemical protein fractionation (salting out), giving active proteins in the precipitate fraction (ASP); (iii) a high-performance liquid chromatography (HPLC) separation by size-exclusion gel filtration to obtain a limited number of protein peaks (P1-P5). This procedure allowed us to obtain five different *C. reniformis* protein fractions, shown in the chromatogram of Figure 2A, with decreasing molecular weight, named P1 to P5, and in the range of: P1 480–250 kDa, P2 250–130 kDa, P3 130–75 kDa, P4 75–35 kDa and P5 35–15 kDa. As shown in the MTT tests in Figure 3, only P4 and P5 HPLC fractions retained a significant cytotoxic effect, thus, these two peaks were processed for the proteomic analysis. Both HPLC fractions were therefore dried and subsequently subjected to two-dimensional (2D) electrophoretic analysis. The results are summarized in Figure 2B, C where a very similar and well-defined row of spots in the same position in the 2D gels is evident (spots from 1 to 4), suggesting the presence of the same protein in both peaks. More specifically, peak 5 showed a purer composition, whereas peak 4 contained traces of other proteins together with the protein showing a cytotoxic activity. The HPLC analysis is coherent with the MS result, where the chromatogram obtained clearly suggests that peaks 4 and 5 are not completely resolved and the proteins eluted in peak 5 are also present in peak 4 (Figure 2A). The MS analysis of the proteins extracted from the four spots of the 2D gels of peaks 4 and 5 corresponds to a protein that we named chondrosin (see Table 1 and Table 2). The row of spots is considered typical of post-translational modifications like that of different patterns of phosphorylation or glycosylation [20,21]. In all four spots, the same peptides were identified. Matches between the MS data and *C. reniformis* transcriptome database allowed us to identify a putative sequence corresponding to the one showed in Figure 2D. The MS analysis clearly identified the primary sequence of the protein, and the data mining of *C. reniformis* transcriptome database allowed to identify a full-length cDNA encoding for a 199-aa polypeptide, with a signal peptide of 21 aa (Figure 2D, underlined in red). The cleavage site of the signal peptide is positioned between amino acids (aa) AEA and SK, with 0.963% of probability (see methods). The mature protein consists of 178 aa (Figure 2C in black), with a theoretical Molecular weight (MW) of 19611.12 and an Isoelectric point (IP) of 5.11. MS analysis confirmed the primary sequence of chondrosin with a 65.17% of coverage (Figure 2D, MS-identified peptides highlighted in yellow). The uncovered part of the sequence is probably the site of post-translational modifications. The in silico predicted structure analysis (Figure 2E) evidenced a clear homology with the *N*-terminal region of the ryanodine receptor/channel and with a small region found in the defensin beta sheet domain (Supplementary Materials, File S1). Many toxins contain defensin-like domains conjugated with different types of protein domains. The genetic toxin evolution, in these cases, starts from a gene encoding a non-toxic protein subjected to mutation by duplication and/or exon shuffling phenomena, and/or specific point mutations, to give rise to the final toxic protein [22,23]. The absence of *C. reniformis* genome database does not allow more specific considerations about the genetic origin of chondrosin, but the presence of this short defensin domain might be explained by a related evolutionary process. More intriguing and peculiar is the presence of a larger domain with high structural homology with the ryanodine receptor. To the best of our knowledge, there has been no previous report for protein toxins, and the hypothesis of action discussed in the following sections is challenging.

### 2.3. Anti-Tumour Activity of Protein Purified Fractions Derived from C. reniformis CE

The cytotoxic action of CE was better characterized by purification of the active compounds responsible of the observed effects. At each purification step, the total protein content of each fraction was quantified (see Section 4), and the increasing concentration of the active protein(s) was monitored by testing its(their) cytotoxic/anti-tumour activity using the MTT test (Figure 3) on normal human dermal fibroblasts (NHDF) and on four cancer cell lines at 24 h incubation at various dilutions (1 to 100 μg/mL total protein content). The cytotoxicity test on NHDF at 24 h incubation showed no effect on the cell viability with respect to control cells in the presence of the DE fraction, at all concentrations tested (Figure 3A). At the same time the ASP fraction showed a weak cytotoxicity only at the highest concentrations (18.5 ± 7.5 and 14.7 ± 7.1% cell death at 100 and 10 μg/mL compared to the control, respectively, *p* > 0.05 for both). Finally, whereas P1 to P3 HPLC purified fractions exhibited no sign of toxicity in all cell lines tested from normal fibroblasts to cancer cell lines (data not shown), P4 and P5 fractions showed a significant cytotoxicity in all types of cells. In particular, in NHDF cells, both P4 and P5 fractions manifested a significant cytotoxicity at the highest and the middle concentrations tested (100 and 10 μg/mL, respectively), namely 73.1 ± 3.7 and 42.1 ± 6.9% cell death were observed for P4 (*p* < 0.0001 vs. C for both bars, respectively), while 81.1 ± 6.3 and 44.2 ± 8.2% cell death were recorded for P5 (*p* < 0.0001 vs. C for both bars, respectively). Finally, at the lowest concentration (1 μg/mL) both P4 and P5 showed no sign of cytotoxicity on NHDF cells. Subsequently, the cytotoxic test was performed on four different cancer cell lines: L929 murine fibroblasts (tumorigenic in immunocompromised mice), RAW 264.7 (murine leukemic macrophages), MDA-MB-468 (human breast carcinoma) and HeLa (human cervical carcinoma). L929 and RAW 264.7 cells showed the highest sensitivity to the cytotoxic effects of the *C. reniformis* protein fractions, whereas the MDA and HeLa cells were the less sensitive, although also in these cell lines a significant effect was observed. Concerning L929 cells, all *C. reniformis* protein fractions i.e., from DE to ASP and from P4 and P5 showed a relevant cytotoxic effect (Figure 3B). DE fraction was dramatically effective both at 100 and 10 μg/mL concentrations while at 1 μg/mL concentration cell death percentage reached 82.8 ± 3.3% compared to control cells (*p* < 0.0001). A similar pattern was observed also for ASP toxicity test where the highest concentrations tested (100 and 10 μg/mL) showed a 94.9 ± 0.5 and 84.7 ± 2.3% cell death while the lowest (1 μg/mL) a 30 ± 4.2% mortality compared to the control (*p* < 0.01). Finally, the cytotoxicity of P4 and P5 HPLC fractions was very high at the highest and middle concentrations tested (87.7 ± 4.2 and 74.6 ± 1.2% for P4 and 80.2 ± 3.1 and 74 ± 2.5% for P5, respectively, *p* < 0.0001 vs. C for both concentrations and both purified peaks) but completely ineffective at the lowest concentration. RAW 264.7 murine leukemic macrophages revealed the highest sensitivity to the *C. reniformis* protein fractions cytotoxic effect. In fact, all fractions from DE to ASP and to P4 and P5, at the highest and middle concentrations tested, showed a cell mortality close to 100% (Figure 3C) and the lowest concentration was still able to significantly affect cell viability vs. control, with a mortality of 76.7 ± 4.4% for DE 1(*p* < 0.0001), 53.9 ± 10.5% for ASP 1 (*p* < 0.0001), 18 ± 9.5% for P4 1 (*p* < 0.05) and 36.7 ± 9.3% for P5 1 (*p* < 0.0005). In MDA-MB-468 breast carcinoma cell line, a different behaviour was observed (Figure 3D). The *C. reniformis* protein fractions never reached a very high rate of mortality, except maybe for DE, ASP and P4 at 100 μg/mL where 79.7 ± 2.5%, 44.5 ± 6.1% and 54 ± 5.9% cell death were reached, respectively (*p* < 0.0001 vs. C for all conditions). Conversely, all the other tested concentrations showed a lower cell death varying from 30–35% yet significantly higher than the control (P5 all concentrations, P4 10 μg/mL, *p* < 0.005) to only 20% (DE 10 and 1 μg/mL, P4 1 μg/mL, *p* < 0.005). Similarly, HeLa cells (Figure 3E) showed a higher rate of mortality only at the highest concentration tested, with a percentage of cell death of 59.4 ± 3.8% for DE, 36.7 ± 1.8% for ASP, 65.7 ± 4.3% for P4 and 51.9 ± 8.1% for P5 compared to C (*p* < 0.005, for all bars), while at the middle and the lowest concentrations cell death rates were around 47.6 ± 2.1% and 29.7 ± 10.7% for DE, 45.6 ± 3.3% and 34.7 ± 10.4% for ASP, 30% for P4 and 35.7 ± 1.3% and 41.8 ± 8.1% for P5, respectively (*p* < 0.05 vs. C for all bars).

Overall, these data indicate that there is a higher cytotoxicity of the *C. reniformis* protein fractions to the cancer cell lines compared to the primary normal cells (Figure 2B–E vs. 2A) with a respective EC_50_ calculated for the P4 and P5 purified fractions of 17.8 and 14.6 μg/mL for NHDF, 3.56 and 4.9 μg/mL for L929, 1.99 and 1.12 μg/mL for RAW 264.7, 59.88 μg/mL for P4 in MDA-MB-468 and 17.7 μg/mL for P4 in HeLa cells. The EC_50_ of the P5 fraction for MDA and HeLa cells was not possible to calculate. Thus, we can conclude that the identified protein chondrosin, present in P4 and P5 fractions, seems to exert an anti-tumour effect on two of the four cancer cell lines (L929 and RAW 264.7) and a cytotoxic/anti-proliferative effect on the other two cell lines (MDA-MB-468 and HeLa). Furthermore, a different sensitivity to the *C. reniformis* anti-tumour protein chondrosin in the four cancer cell lines can be envisaged, probably due to some differences in the chondrosin specific interaction with these cells, as further discussed in Section 2.4. In particular, the two murine tumorigenic cell lines (panels B and C) showed the highest sensitivity to *C. reniformis* protein fractions, with the hematologic cancer cell line being the most affected of all, while the two human carcinomas (panels D and E) demonstrated a higher resilience to the cytotoxic effect, even at the lowest protein fraction concentration (1 μg/mL), cell viability was always significantly lower than the untreated control (in MDA between 20 and 30% less cells, in HeLa between 30 and 40% less cells). These numbers could indicate that while a strong cell death mechanism could operate in L929 and RAW 264.7 murine cells, the effect in the human carcinoma cell lines could be more cytostatic, resulting in a lower cell death rate and a slow-down of cell growth whose cell death never reached the values of the untreated controls.

### 2.4. Mechanisms of Toxicity of Chondrosin on Tumour Cells

#### 2.4.1. Necrosis and Apoptosis Assessment

**Figure 4:**
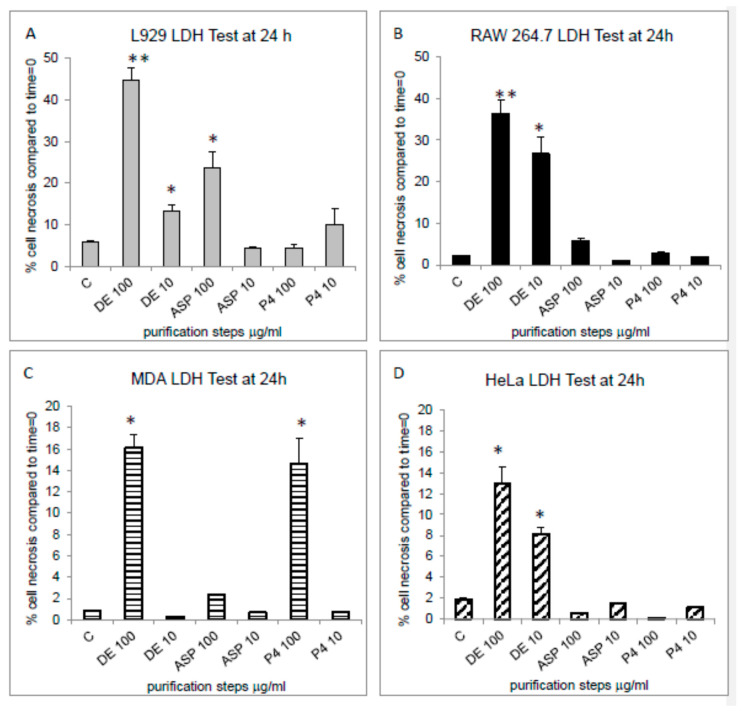
Cell necrosis assessment. (**A**) The percentage of necrotic cells measured by quantification of LDH release in the cell medium at 24 h in L929 murine tumorigenic fibroblasts after incubation in the presence or absence (Control, C) of DE, ASP and P4 (100 and 10 μg/mL, respectively). Results are expressed as percentage of dead cells with respect to the number of seeded cells at time = 0 and are the mean ± S.D. of three experiments performed in quadruplicate. Asterisks indicate significance in paired Tukey test (ANOVA, *p* < 0.00001; Tukey vs. C: ** *p* < 0.001, * *p* < 0.05, respectively). (**B**) The percentage of necrotic cells measured in the same conditions as (A) in RAW 264.7 murine leukemia cells. Asterisks indicate significance in a paired Tukey test (ANOVA, *p* < 0.00001; Tukey vs. C: ** *p* < 0.001, * *p* < 0.05, respectively). (**C**) The percentage of necrotic cells measured in the same conditions as (**A**) in MDA-MB-468 human breast carcinoma cells. Asterisks indicate significance in paired Tukey test (ANOVA, *p* < 0.001; Tukey vs. C: * *p* < 0.05). (**D**) The percentage of necrotic cells measured in the same conditions as (A) in HeLa human cervical carcinoma cells. Asterisks indicate significance in a paired Tukey test (ANOVA, *p* < 0.001; Tukey vs. C: * *p* < 0.05).

**Figure 5:**
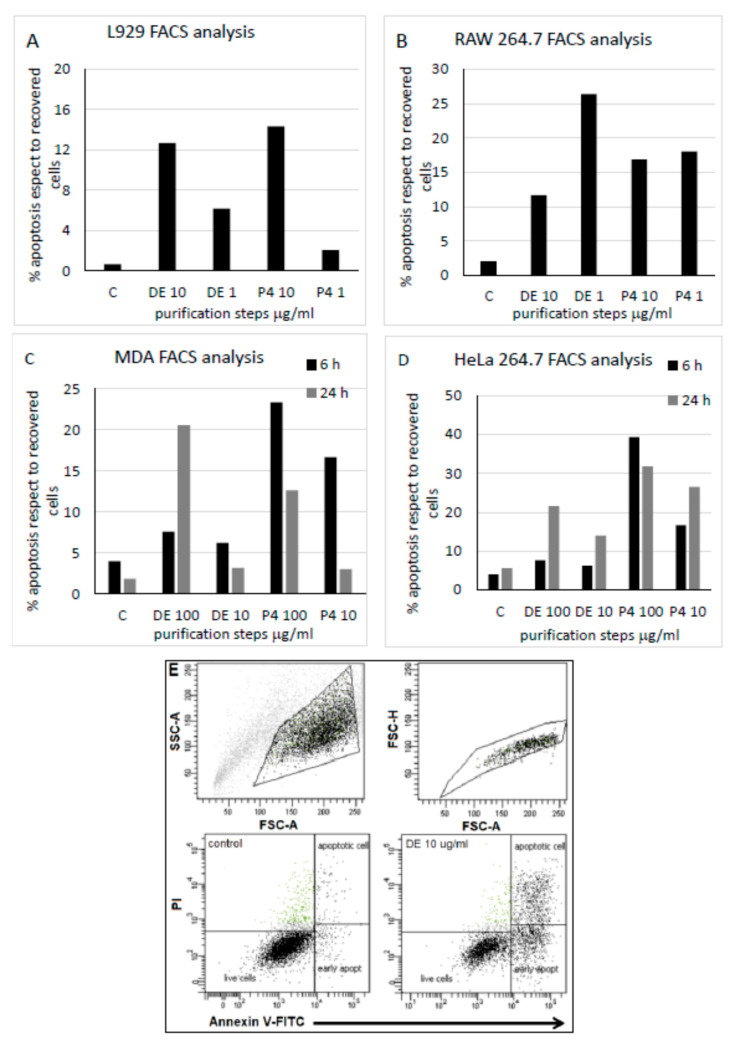
Cell apoptosis assessment by FACS analysis. (**A**) The percentage of annexin-positive, apoptotic cells measured by FACS analysis in trypsin-detached L929 murine tumorigenic fibroblasts after 6 h incubation in the presence or absence (**C**) of DE and P4 (10 and 1 μg/mL, respectively). Results are expressed as percentage of positive apoptotic cells compared to the total number of still living cells retrieved from the plate by trypsin detachment and are representatives of one of the two FACS acquisition experiments performed in duplicate. (**B**) The percentage of annexin-positive, apoptotic cells measured in the same conditions as cells in panel (**A**) in RAW 264.7 murine leukemia cells. (**C**) The percentage of annexin-positive, apoptotic cells measured by FACS analysis in trypsin-detached MDA-MB-468 cells after 6 (black bars) or 24 h (grey bars) incubation in the presence or absence (**C**) of DE and P4 (100 and 10 μg/mL, respectively). Results are expressed as percentage of positive apoptotic cells compared to the total number of still living cells retrieved from the plate by trypsin detachment and are representatives of one of the two FACS acquisition experiments performed in duplicate. (**D**) The percentage of annexin-positive, apoptotic cells measured in the same conditions as cells in panel (**C**) in HeLa human cervical carcinoma cells. (**E**) Representative image of FACS analysis raw data obtained in annexin/PI detection on HeLa cells at 6 h incubation in the absence (control) or presence of 10 μg/mL DE.

**Figure 6:**
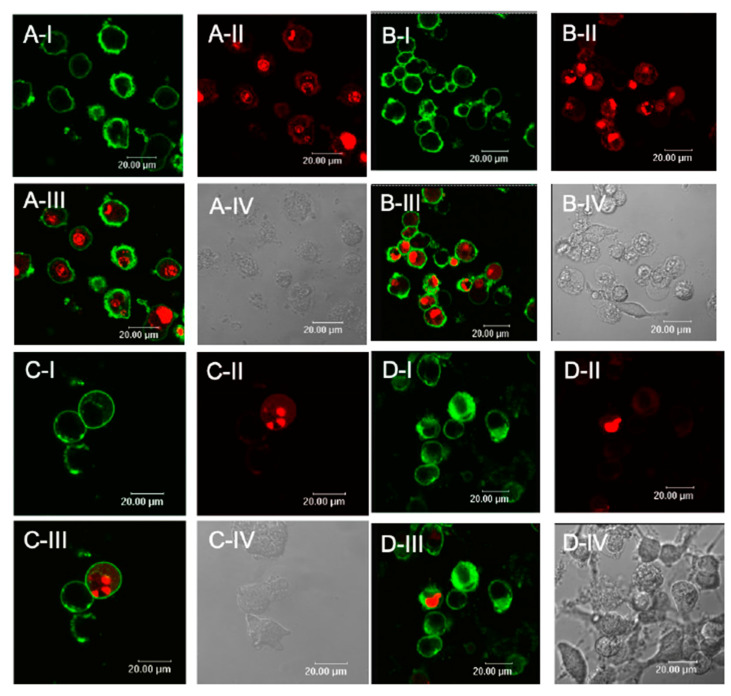
Cell apoptosis assessment by confocal microscopy analysis. (**A**) Visualization by confocal microscopy analysis (HCX PL APO CS 63.0 × 1.40 oil objective, 2 × digital zoom) of annexin-positive (green fluorescence) and/or propidium iodide-positive (red fluorescence) cells in L929 murine tumorigenic fibroblasts after 6 h incubation in the presence of 100 μg/mL P4. Panel I: green channel, panel II, red channel, panel III: superimposed green/red channels, panel IV: phase contrast. The white bar spans 20 μm. (**B**) Visualization of RAW 264.7 murine leukemia cells measured in the same conditions as cells in panel (**A**). (**C**) Visualization of MDA-MB-468 human breast carcinoma cells measured in the same conditions as cells in panel (**A**). (**D**) Visualization of HeLa human cervical carcinoma cells measured in the same conditions as cells in panel (A).

To investigate which type of cell death the four cancer cell lines were undergoing, two types of analyses were performed: the Lactate Dehydrogenase (LDH) assay, quantifying the enzyme leakage in the cell medium, in order to evaluate the level of cell death by necrosis in the cultures, and the annexin/propidium iodide positivity assay, analysed both by cytofluorimetry (FACS analysis), and by confocal microscopy in order to establish the level of apoptosis of the cancer cells. The LDH assay performed after 24 h of incubation revealed a certain rate of necrosis in the four cancer cell lines (Figure 4), but mainly in the presence of the DE fraction and not in the further purified fractions, and especially in the two murine cancer cell lines (L929 and RAW 264.7, panels A and B, respectively). In detail, L929 cells showed a significant cell necrosis in the DE fraction at both concentrations tested compared to control cells (44.6 ± 3.1% and 13.3 ± 1.4% at 100 and 10 μg/mL, *p* < 0.001 and *p* < 0.05, respectively), and also in the ASP fraction at the highest concentration (23.5 ± 3.8%, *p* <0.05), while in the P4 fraction the percentage of necrosis was always below 10% and comparable to the control. RAW 264.7 cells showed a comparable behaviour with a significant necrosis with respect to that of the control, observable only in the DE fraction (36.2 ± 3.4% and 26.6 ± 4%, at 100 and 10 μg/mL, *p* < 0.001 and *p* < 0.05, respectively), while the ASP and P4 fractions always showed a necrosis percentage below 10% and comparable to that of the control. In MDA cells (Figure 4C) necrosis was only observable at the highest concentration of DE and P4 fractions in the range of 15% for both, compared to the control (*p* < 0.05), and finally in HeLa cells, the only protein fraction causing a slight but measurable rate of necrosis was the DE at both concentrations tested (13 ± 1.6% and 8.1 ± 0.6%, at 100 and 10 μg/mL, respectively, *p* < 0.05). Overall, the two human cell lines showed a very low level of necrosis, although still measurable, in their cultures. These data indicate that: (i) in the DE protein fraction there is a multifactorial cytotoxic activity provoking cell death partly by necrosis, as documented in Figure 4, but not only, and this is especially visible at 100 μg/mL DE protein fraction concentration where the rate of cell death measured by the MTT test (Figure 3) is significantly higher compared to the percentage of necrosis at the same time point (24 h) in all cancer cell lines; (ii) in the further purified *C. reniformis* protein fractions, namely the ASP and the P4, the necrotic component of the protein extract has been eliminated by the purification steps, and the still high rate of cytotoxicity measured in these fractions in all cancer cell lines at 24 h (MTT test in Figure 3) is probably due to an apoptotic mechanism. To test this hypothesis, the annexin/propidium iodide staining was used to evaluate the apoptotic state in all cancer cell lines and the cultures were analysed by both Fluorescence-activated cell sorting flow cytometry (FACS) and confocal microscopy for a quantitative and qualitative assessment, respectively. In particular, the FACS analysis was performed at 6 and 24h on the four tumour cell lines on the cells recovered after trypsin detachment from the wells where the treatment with the DE or P4 was performed. Thus, the results refer to the percentage of apoptotic cells observed in the population of cells still attached to the plate and, therefore, still alive in the plate. Results are shown in Figure 5 and exemplify one of the three experiments performed on the four cell lines. Concerning L929 (panel A) and RAW 264.7 cells (panel B) only the 6 h endpoint is shown since at 24h it was not possible to retrieve a sufficient number of manageable cells from the plates to perform the analysis, and for the same reason the concentrations of protein fractions used for these cells were of 10 and 1 μg/mL for both DE and P4 fractions. In both cell lines it was possible to observe, at the only time point analysed, a significant number of apoptotic cells (the percentage is the sum of early + late apoptotic) with respect to all the cells retrieved from the plates. The highest percentage of apoptotic cells in L929 cell line was observed in the sample treated with 10 μg/mL P4 (14.3%) and in the RAW 264.7 cell line treated with 1 μg/mL DE (26.4%), although in these cells also the P4 treatment, both at 10 and 1 μg/mL, showed a significant percentage of apoptosis relative to the total number of cells retrieved from the plate (18.8% and 18.1%, respectively). Interestingly, also in MDA (panel C) and HeLa cells (panel D–E), although the *C. reniformis* protein fractions were less cytotoxic (see Figure 3), it was possible to observe a significant number of apoptotic cells both at 6 and 24 h at 100 and 10 μg/mL concentrations for the DE and P4 treatments. In particular, in both MDA and HeLa cells, the highest percentage of apoptotic cells was observed in the P4 treatment at the highest concentration after 6 h incubation (23.3% and 39.3%, respectively), while at 24 h the percentage of apoptosis in the same sample was lower in both cell lines (12.6% and 31.8%, respectively). Furthermore, in the same cell lines the second highest apoptotic percentage was obtained by treatment with the DE at 100 μg/mL for 24 h, where in both cell lines the level of apoptosis was around 20% with respect to all cells retrieved from the plates (panels C, D and E). These data were also confirmed by a confocal microscopy analysis of the four cell lines, after treatment with P4, by annexin/propidium iodide staining of cells (Figure 6). In particular, L929 (panels A) and RAW 264.7 cells (panels B) images were acquired after 6 h treatment with 10 μg/mL P4. In both cell lines it was possible to observe numerous groups of cells showing signs of both early (only green positivity) and late (concomitant green/red positivity) apoptosis (panels A-III and B-III, respectively), being the second more prevalent than the first (i.e., more green/red cells than only green in both cell lines). Conversely, both in MDA (panels C) and HeLa cells (panels D) treated with 100 μg/mL P4 for 6 h, early apoptotic cells (only green positivity) were more prevalent than late apoptotic (concomitant green/red positivity) cells at this time point (panels C-III and D-III, respectively). Altogether, these data indicate that a significant phenomenon of apoptosis is observable in the four tumor cell lines treated with the *C. reniformis* protein fractions. Thus, we can infer that the cytotoxicity of chondrosin, a new protein identified as the main component of the P4 and P5 fractions, is likely due to a mechanism promoting apoptosis in the affected cells.

#### 2.4.2. Cytosolic Calcium Rise and Reactive Oxygen Species (ROS) Production in Challenged Cells

**Figure 7:**
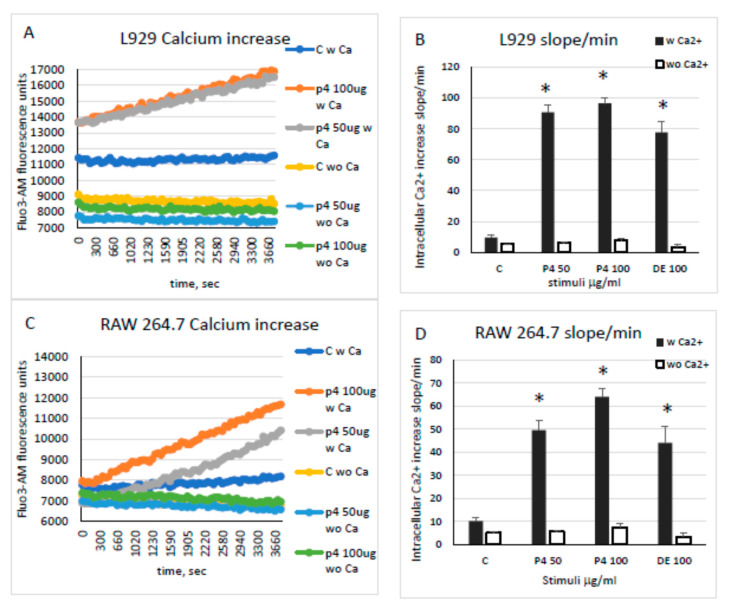

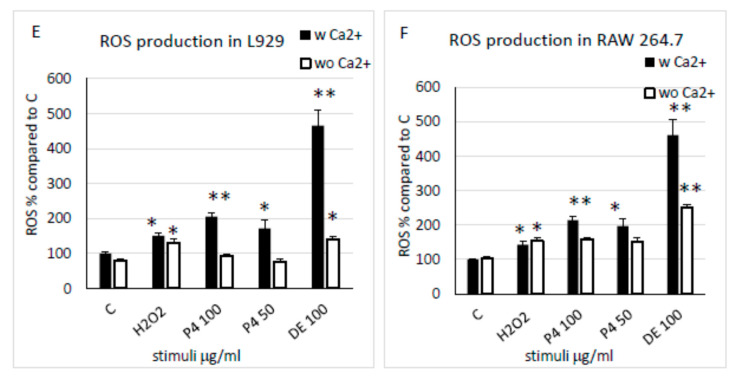
Intracellular Ca^2+^ and ROS quantification by fluorometric analysis. (**A**) Intracellular Ca^2+^ concentration increase evaluation over time in Fluo3-AM-loaded L929 murine tumorigenic fibroblasts challenged without (C w Ca) or with P4 at 100 and 50 μg/mL immediately before starting the fluorometric acquisition at 480/520 ex/em. The experiments were performed in the presence (w Ca) or absence (wo Ca) of Ca^2+^ in the extracellular medium. Each coloured line represents the recording at every minute of a single well of a 96-well plate for 1 h. Every line is representative of two experiments performed in triplicate. (**B**) Calculated slope/min of each intracellular Ca^2+^ increase in 1 h time interval (from experiment in **A**) for each condition of analysis performed in triplicate (control: C, P4 50 and 100 μg/mL, DE 100 μg/mL) in L929 murine tumorigenic fibroblasts. Slope/min was calculated from acquisitions either in the presence (black bars) or absence (white bars) of Ca^2+^ in the extracellular medium. Asterisks indicate significance in Tukey test (ANOVA for black bars *p* < 0.000001; Tukey vs. C, * *p* < 0.0001). (**C**) Intracellular Ca^2+^ concentration increase evaluation over time in RAW 264.7 murine leukemia cells measured under the same conditions as cells in panel (**A**). (**D**) Calculated slope/min of intracellular Ca^2+^ increase in RAW 264.7 murine leukemia cells was measured under the same conditions as cells in panel (**B**). Asterisks indicate significance in Tukey test (ANOVA for black bars *p* < 0.000001; Tukey vs. C, * *p* < 0.0001). (**E**) Intracellular ROS production measured by DCF fluorometric analysis in L929 murine tumorigenic fibroblasts incubated for 2 h with 200 μM H_2_O_2_ (positive control) or with 50 or 100 μg/mL P4 or 100 μg/mL DE, in the presence (black bars) or absence (white bars) of Ca^2+^ in the extracellular medium. Results are expressed as percentages of ROS production respect to the control (**C**) and are the mean ± SD of three experiments performed in quadruplicate. Asterisks indicate significance in Tukey test (ANOVA for black bars *p* < 0.000001; Tukey vs. C, ** *p* < 0.0001, * *p* < 0.01, respectively; ANOVA for white bars *p* < 0.000001, Tukey vs. C, * *p* < 0.01). (**F**) Intracellular ROS production measured by DCF fluorometric analysis in RAW 264.7 murine leukemia cells measured in the same conditions as cells in panel (**E**). Asterisks indicate significance in the Tukey test (ANOVA for black bars *p* < 0.000001; Tukey vs. C, ** *p* < 0.0001, * *p* < 0.01, respectively; ANOVA for white bars *p* < 0.00005, Tukey vs. C, ** *p* < 0.0001, * *p* < 0.01, respectively).

Two main signalling stress mediators, and eventual inducers of cell death and apoptosis, are represented by a sustained cytosolic Ca^2+^ rise and reactive oxygen species (ROS) production [24]. The duration and intensity of these well-known stress signals are the key factors for an adequate cell response to dangers the cells are facing or, if too prolonged in time and/or too severe in its intensity, are the means to induce cell death, indicating the inability of the cells to overcome the challenge they are facing. Due to the similarity shown by the new identified protein chondrosin to the ryanodine receptor/channel (see Figure 2D,E), we decided to investigate the effect of chondrosin on the intracellular Ca^2+^ concentration rise by using a calcium-sensitive dye (Fluo3-AM) and fluorometric analysis (Figure 7A–D). Both fluo3-AM-loaded L929 and RAW 264.7 cells (panels A,B and C,D, respectively) were challenged with different P4 and DE concentrations (50 and 100 μg/mL and 100 μg/mL, respectively) and intracellular fluorescence was then monitored for 1 h total acquisition. Panels A and C show representative intracellular Ca^2+^ rise curves in single wells challenged with the two different P4 concentrations in the two cell lines (L929 and RAW 264.7, respectively) in the presence or absence of extracellular Ca^2+^ in the cell culture medium; conversely panels B and D show the slope/min ± SD for each stimulus calculated from the curves shown in panels A and B, respectively. In the presence of extracellular Ca^2+^ in the culture medium, both P4 and DE were able to induce a slow but unrelenting cytosolic Ca^2+^ rise in L929 and RAW 264.7 cells with respect to unchallenged control cells (“w Ca” lines in panels A and C, and black bars in panels B and D, respectively, ANOVA *p* < 0.000001 for both cell lines). Conversely, in the absence of extracellular Ca^2+^, the intracellular rise of this important mediator was completely abolished over time after stimulation with both P4 and DE in both L929 and RAW 264.7 cell lines (“wo Ca” lines in A and C, and white bars in B and D, respectively). These data clearly indicate that chondrosin, in the P4 enriched fraction, is able to induce a significant intracellular Ca^2+^ rise by extracellular Ca^2+^ entry into the cytoplasm from the cell membrane probably by activating a plasma membrane Ca^2+^-receptor/channel. Our hypothesis is focused on the possible activation, by direct interaction with chondrosin, of Cav 1.3 calcium channel, a L-type voltage-dependent ion channel, also recently described on the plasma membrane of RAW 264.7 cells [25]. The opening of this channel allows the extracellular Ca^2+^ entrance. Recently, in hippocampus neurons it has been indeed demonstrated the activation of this channel via physical interaction with the ryanodine receptor, which chondrosin closely resembles, with the consequent entrance of Ca^2+^ from the synaptic area [26].

Since another important stress signal activating cell death pathways is intracellular ROS production, directly or indirectly stimulated by the extracellular stimulus/danger or by the Ca^2+^ second messenger rise, respectively [24,27], also this parameter was investigated in L929 and RAW 264.7 cells by using the ROS sensitive dye 2′,7′-dichlorodihydrofluorescein diacetate (DCF) and fluorometric analysis (Figure 7E,F). The analysis was performed either in the presence or absence of extracellular Ca^2+^ in the cell medium. In both cell lines (L929 panel E and RAW 264.7 panel F, respectively) it was possible to observe a significant intracellular ROS production compared to the control, in the presence of extracellular Ca^2+^ (black bars, ANOVA *p* < 0.000001 for both cell lines), after 2 h incubation with P4 (50 and 100 μg/mL) and DE (100 μg/mL), similar to (P4) or significantly higher than (DE) the 200 μM H_2_O_2_ positive control stimulus. Conversely, in the absence of extracellular Ca^2+^ (white bars), a significant inhibition of ROS production after P4 and DE challenge was observed in both cell lines. In contrast, the positive H_2_O_2_ control remained essentially unaffected by the absence of the extracellular Ca^2+^. These data indicate that while cytosolic ROS production in H_2_O_2_-challenged L929 and RAW 264.7 cells is not influenced by the extracellular Ca^2+^ concentration, in the case of P4 and DE challenged cells and, thus, in the presence of chondrosin, extracellular Ca^2+^ entry in the cells is totally (L929, panel E) or partially, but significantly (RAW 264.7, panel F), responsible for the consequent ROS production, depending on the cell line. This also indicates that cell type-specific responses and a different sensitivity to the stimulus can be observed in chondrosin-challenged cells. From these data we can fairly conclude that extracellular Ca^2+^ entry is a triggering signal for ROS production in chondrosin-challenged cells and that the significant cytosolic rise of these two important mediators is likely the main reason for the subsequent cytotoxic-apoptotic outcome in the tumour cells. Furthermore, from the analysis of Ca^2+^ and ROS data in DE-challenged cells, some other conclusions can be drawn on the cytotoxic activity of the *C. reniformis* protein-containing fractions. In particular it is to note that, in terms of signal intensity: (i) the intracellular Ca^2+^ rise in DE-challenged cells is of the same order of magnitude as the P4 stimulus (Figure 7 panels A–D), an effect likely due in both fractions to the chondrosin; and (ii) in the case of ROS intracellular production DE-challenged cells showed more than a two-fold increase compared to the P4 stimulus. This last result suggests the presence of a multifactorial component in the DE fraction, where the ROS increase is generated by the sum of a protein component causing the extracellular Ca^2+^ entry (very likely chondrosin) and of the action of another protein component probably directly stimulating a ROS production independent from extracellular Ca^2+^ entry. This is especially visible in the RAW 264.7 cell line (panel F). This significant difference in ROS production observed in the DE fraction compared to the P4 fraction, could also help to explain the higher rate of necrosis observed in DE-treated cells with respect to P4 (Figure 4), probably due to a more direct cytotoxic effect of the ROS overproduction in the first case relative to the second. The DE component responsible for the ROS overproduction and the higher rate of necrosis is lost in the P4 purified fraction enriched in chondrosin, where the ROS production is significantly lower and probably mainly due to the chondrosin action, through the stimulation of the extracellular Ca^2+^ entry. This allows to infer that, in the P4-treated cells, chondrosin exerts its action mainly by promoting apoptosis and in lesser extent, by a direct cytotoxic effect, contrary to what likely happens in the cells treated with the less purified DE fraction.

## 3. Conclusions

In this work we identified a new type of cytotoxic protein from the marine sponge *C. reniformis*, with unique features. Chondrosin is likely secreted to the surrounding environment when the sponge is subjected to a strong stress or after wounding. The structure of this toxin shows a domain with intriguing structural homology with the *N*-terminal region of the ryanodine receptor/Ca^2+^ channel. Furthermore, in this toxin, a short domain with high homology to defensins is also present. The presence of defensins regions and domains is known in many toxins of protein origin from arthropods to reptiles, while the toxic action of the N-terminal region of the ryanodine receptor/channel domain is observed for the first time. We demonstrated that the toxic action is mediated by an extracellular calcium intake followed by a cytoplasmic ROS overproduction, occurring mainly in some tumour cell lines (i.e., RAW 264.7 and L929). We suggest that this selective action could be related to the expression of Cav 1.3 ion voltage-dependent channel on the cell membranes of the affected cells. Recent publications have shown a physical interaction of the ryanodine receptor with the Cav 1.3 membrane receptor, with consequent extracellular calcium entrance. Due to the high similarity of chondrosin to the ryanodine receptor, this could also be the mechanism of action of the newly identified toxic protein from the marine sponge *C. reniformis*.

## 4. Materials and Methods

*Chemicals:* All reagents were acquired from Sigma-Aldrich (Milan, Italy), unless otherwise stated.

### 4.1. Preparation of Crude Extracts (CE) of C. reniformis

Specimens of *C. reniformis* were collected in the area of the Portofino Promontory (Liguria, Italy) at depths of 10–20 m and transferred to the laboratory in a thermic bag. During the transport, the temperature was maintained at 14–15 °C. A short-term animal housing, of no more than 24–48 h, was performed as described previously [28]. In particular, the sponges were stored at 14 °C in 200-L aquaria containing natural sea water collected in the same area of the Portofino Promontory with a salinity of 3.7% and equipped with an aeration system.

The sponge samples (20–40 g wet weight) were washed with sterile artificial sea water (ASW) and then were cut into very small pieces using a sterile scalpel and kept in a small volume of sterile TRIS-HCl 50 mM pH 8.0 containing an anti-protease mix (pepstatin and leupeptin 1mM) to protect the proteins from the action of eventual proteases released during squeezing. The fragments were vigorously squeezed with both a sterile gauze and a garlic squeezer in order to collect the liquid constituting a crude extract (CE). The procedure was performed at 0 °C on ice, to avoid the possible degradation of the CE components. The CE, with a final volume in the range of 30–50 mL depending on the dimensions of the sponge, was then centrifuged at 8000× *g* for 10 min at 4 °C to eliminate sponge debris and to obtain a clear CE.

#### 4.1.1. Chemical Nature Assessment of CE Cytotoxic Activity

In order to investigate the chemical nature of the sponge cytotoxic activity, the CE was subjected to various treatments to assess the thermal stability, the molecular weight range and the sensitivity to trypsin digestion of the unknown compound(s). Following these steps, the cytotoxic activity was evaluated on the L929 cell line via the MTT test as described in Section 4.3.1. To assess the thermal stability of the cytotoxic component of the clear CE, the extract was heated for 10 min in a heat block at 100 °C and its activity was compared with an untreated sample. To establish the molecular range of the compound(s) involved in the cytotoxic activity, the clear CE was subjected to a dialysis step for 24 h, at 4 °C, against 100 volumes of 50 mM Tris-HCl buffer at pH 8.0, the cut-off of the dialysis membrane was 10,000 kDa to get rid of low-molecular weight sponge metabolites and mainly retaining the protein fraction obtaining a dialysed extract (DE). After that, in order to confirm the protein nature of the sponge cytotoxic activity, the DE was digested with porcine trypsin (Sigma-Aldrich, Milan) at 1:20 (*w:w*) or 1:50 (*w:w*) for 24 h at 37 °C. To confirm the trypsin digestion activity 20 μg of the undigested DE sample and 20 μg of the digested, samples were subjected to SDS-Page analysis on a 12% acrylamide gel. At the end of the run the gel was fixed in 40% ethanol, 10% acetic acid for 60 min and finally stained with colloidal Coomassie blue [29]. The cytotoxic activity of the trypsin-digested and undigested DE was then assessed by MTT test on the L929 cell line as described in Section 4.3.1.

#### 4.1.2. Salting out of DE Proteins

The dialysed extract (DE) was concentrated to a final volume of 10–15 mL by centrifugation at 3500× *g*, at 4 °C, on “Macrosep® Advance Centrifugal Devices With Omega™ Membrane 10K” (PALL Corporation, NY, USA) with a 10,000 kDa cut-off. Protein concentration was then quantified by the Bradford method [30].

The following purification step was based on the salting out method by ammonium sulphate (AS) precipitation of protein fractions. In particular, a solution of 70% saturated AS was used to separate two protein fractions: a precipitated fraction (ASP) and a soluble fraction (ASS). The DE was diluted to a final protein concentration of 5 mg/mL in TRIS-HCl 50 mM pH 8.0, and was precipitated by putting the tube containing the solution in ice and slowly adding (drop by drop) a solution of concentrated ice-cold AS to finally reach a 70% of salt saturation of the entire solution. The slow addition was completed in 1 h and, afterwards, the 70% AS saturated solution was left to slowly stir maintaining the tube containing the solution in ice for further 2 h to let proteins precipitate. The suspension was then centrifuged at 5000× *g*, at 4 °C, for 30 min to recover a soluble protein fraction from the supernatant (ASS) and a precipitated protein fraction from the pellet (ASP). The ASS was again concentrated to a few mL by centrifugation at 4 °C as already described (Macrosep® Advance Centrifugal Devices cut-off 10,000 kDa), while the ASP was resuspended in a small volume (2 mL for each 5 mg of initial protein used for the salting out) of TRIS-HCl 50 mM, pH 8.0, and both ASS and ASP were dialysed against 100 volumes of TRIS-HCl 50 mM, pH 8.0, at 4 °C, to get rid of AS salt excess (cut-off 10,000 kDa). The protein quantification by Bradford assay was again performed on both fractions to calculate protein recovery.

#### 4.1.3. HPLC Protein Purification

The protein separation by molecular weight gel filtration of the ASP purification step was carried out in a HPLC Agilent 1260 system (Agilent Technologies, Palo Alto, CA, USA). The separation was performed on an Agilent SEC-5 column (30 cm × 7.8 mm I.D., 5 μm) equipped with an Agilent SEC-5 guard column (5 cm × 7.8 mm I.D.) at 1 mL/min for 25 min in isocratic mode. Absorbance of the eluted fractions was monitored at 220 nm. The eluent was 0.1 M phosphate buffer, pH 6.0, with 0.1 M sodium sulphate buffer. Five fractions were collected from the column elution corresponded to five main peaks:

P1: from min 6 to min 8;

P2: 8 to 9.5 min;

P3: 9.5 to 10.5 min;

P4: 10.5 to 11.5 min; and

P5: 11.5 to 13.5 min.

Multiple runs were performed, and the five fractions collected were again concentrated by amicon filter centrifugation at 4 °C (cut-off 10,000 kDa, as already described in the previous paragraph) and the protein concentration of each purified fraction (P1 to P5) was then estimated by Bradford assay.

### 4.2. Proteomic Analysis 

#### 4.2.1. Protein Precipitation

A total of 50 μg of proteins were transferred into a Slide-A-Lyzer mini dialysis device (ThermoFischer Scientific, Milan, Italy) at 3500 kDa molecular weight cut-off (MWCO) and dialysed against 1L of deionized water at 4 °C, overnight. Proteins were then precipitated with five volumes of ice-cold acetone (80%) and centrifuged at 14,000× *g* for 30 min. The pellets were washed twice with ice-cold acetone (80%) and resuspended in 5 M urea, 2 M thiourea, 4% 3-[(3-Cholamidopropyl)dimethylammonio]-1-propanesulfonate,(CHAPS), 1% immobilized pH gradient (IPG) buffer, 20 mM 1,4-dithiothreitol (DTT), and traces of Bromophenol blue (BPB).

#### 4.2.2. D Gel Electrophoresis

For the first dimension, 7-cm IPG strips (Bio-Rad Laboratories S.r.l., Segrate, MI, Italy), pH 3–10, were pre-hydrated at 50 V for 12 h in 5 M urea, 2 M thiourea, 4% CHAPS, 1% IPG buffer, 20 mM DTT, and traces of BBF. Isoelectric focusing (IEF) was performed at 300 V for 4 h, followed by a gradient of 300–1000 V in 30 min, 1000–5000 V in 90 min, and then kept at 5000 V for 4h.

The proteins in the strips were reduced for 10 min in 1% DTT in equilibration buffer (50 mM Tris–HCl with pH 8.8; 5 M urea; 2 M thiourea; 30% glycerol; 2% Sodium dodecyl sulfate (SDS); traces of BPB), and then alkylated for 10 min in 2.5% iodoacetamide in the same buffer. The proteins were then separated in a second dimension according to their apparent molecular mass on a 12% acrylamide gel, pH 8.8. After electrophoresis, the gels were fixed in 40% ethanol, 10% acetic acid for 60 min and stained with colloidal Coomassie blue [29].

#### 4.2.3. In-Gel Digestion

The gel protein bands were excised and trypsin digested at 37 °C, overnight according to Shevchenko et al. [31]. Briefly, the gel pieces were de-stained in acetonitrile, reduced using 10 mM DTT in 100 mM ammonium bicarbonate for 30 min at room temperature (RT), and alkylated with 100 mM iodoacetamide in 100 mM ammonium bicarbonate for 30 min at RT in the dark. The tryptic peptide samples were extracted after digestion by sonicating for 10 min at RT, then vacuum dried and stored at −20 °C until MS/MS analysis.

#### 4.2.4. Nano-LC Mass Spectrometry

Tryptic peptides were analysed by nano-HPLC-MS/MS using an Ultimate 3000 nano-HPLC system (managed by CHROMELEON software connected to a Hybrid Quadrupole-Orbitrap mass spectrometer (Q Exactive, Thermo Scientific, Waltham, MA, USA).

The pellets containing the tryptic peptides were resuspended immediately before analysis. The obtained solutions were firstly loaded from the sample loop onto a trapping column (Acclaim PepMap C18; 2 cm × 100 μm × 5 μm, 100 Å-ThermoScientific, Waltham, MA, USA) using the loading eluent (95–5% ACN/H_2_O + 0.05% trifluoroacetic acid) at a flow rate of 5 μL/min for 5 min. The trapping column was then switched in-line with the separation column and the peptides were eluted increasing the organic solvent percentage at a flow rate of 300 nL/min. The separations were carried out at 35 °C using an Easy spray column (15 cm × 75 µm PepMap C18 3 µm Thermo Scientific) and applying a linear gradient from 4% to 95% of solution B (95–5% ACN/H_2_O + 0.08% formic acid) for 55 min.

All the analyses were carried out in the 395–2000 *m/z* range, using a maximal ion injection time of 100 milliseconds. The resolution was set to 70,000 and the automatic gain control was set to 3 × 10^6^ ions. The experiments were performed in data-dependent acquisition mode with alternating MS and MS/MS experiments. The minimum MS signal to obtain MS/MS was set to 500 ions and the most prominent ion signal was selected for MS/MS using an isolation window of 2 Da. The *m/z* values of signals already selected for MS/MS were put on an exclusion list for 5 s using dynamic exclusion. In all cases, one micro-scan was recorded. Collision-induced dissociation (CID) was done with a target value of 5000 ions, a maximal ion injection time of 50 ms, normalized collision energy of 35%. A maximum of 10 MS/MS experiments/MS scans were performed. Raw MS files were processed with the Thermo Scientific Proteome Discoverer software, version 1.4. Peak list files were obtained by the SEQUEST search engine against the *C. reniformis* protein database.

The deduced amino acid sequences database was obtained as described in [4] from *C. reniformis* transcriptome sequencing. This analysis allowed to obtain a series of putative proteins named isotig# (isotigs: contig combinations representing the full mRNAs) where # is a five-digit unique numeric identifier).

The resulting peptide hits were filtered for a maximum 1% FDR (false discovery rate) using the percolator tool. The peptide mass deviation was set to 10 ppm, and a minimum of six amino acids to identify peptides was required. The database search parameters were: mass tolerance precursor 20 ppm, mass tolerance fragment CID 0.8 Da, dynamic modification of deamidation (N, Q), oxidation (M) and static modification of alkylation with 2-Iodoacetamide (IAM) (C). In any case, the option “trypsin with two missed cleavages” was selected.

#### 4.2.5. Protein Sequence Analysis and 3D Modelling

The amino acid sequences of identified proteins were first analysed using the Basic local alignment search tool (BLAST) algorithm at the National Centre for Biotechnology Information (http://blast.ncbi.nlm.nih.gov/Blast.cgi) in order to find identity with previously described proteins. Successively the sequences were analysed using the simple Modular Architecture Research Tool (SMART) program (http://smart.embl-heidelberg.de/) for the identification of conserved domains. The presence of signal peptides was detected using SignalP 4.1 server (http://www.cbs.dtu.dk/services/SignalP/), whereas the theoretical molecular weight and isoelectric point value of the mature protein were obtained using the Expasy tool: https://web.expasy.org/compute_pi/.

Finally, an in silico 3D-structure reconstruction of *C. reniformis* condrosin was performed with Phyre^2^ free software http://www.sbg.bio.ic.ac.uk/phyre2/html/page.cgi?id=index using the normal modelling mode. [32].

### 4.3. Cell Cultures 

The mouse macrophage leukemia cell line RAW 264.7, the mouse fibroblast tumorigenic L929, the MDA-MB-468 human breast carcinoma, the HeLa human cervical carcinoma and normal human primary dermal fibroblasts from adult foreskin (NHDF) were obtained from the American Type Culture Collection (LGC Standards srl, Milan, Italy) and cultured as described in the manufacturer’s instructions. All cell lines, except NHDF cells, were cultured in high glucose Dulbecco’s modified Eagle’s medium (D-MEM) with glutamine (Microtech srl, Naples, Italy), supplemented with 10% foetal bovine serum (Microtech) with penicillin/streptomycin as antibiotics. NHDF primary cells were cultured in D-MEM supplemented with 15% FBS and penicillin/streptomycin. All cells were cultured at 37 °C in a humidified, 5% CO_2_ atmosphere. Experiments were performed in quadruplicate on 96-well plates. 

#### 4.3.1. Cell Toxicity by MTT and LDH Tests

Experiments were performed in quadruplicate on 96-well plates as already described [33]. Briefly, RAW 264.7 macrophages were seeded at 25,000 cells/well, while all other cell lines were seeded at 10,000 cells/well and allowed to adhere overnight. Then the various dilutions of the different purification steps of the RE (final dilutions from 1 to 100 μg/mL of protein concentration) were added to each well and the plates incubated for 24 h at 37 °C. At the end of the experiments cell viability was assayed by MTT test (0.5 mg/mL final concentration) as already reported [34]. For the LDH test, cells were seeded and treated in the same manner as described above in 96-well plates in triplicate and after 24 h incubation the LDH release in the cell media was quantified in each well by the Cytotoxicity LDH Assay Kit-WST (Dojindo EU GmbH, Munich, Germany) following the manufacturer’s instructions. Data are the means ± S.D. of three independent experiments performed in quadruplicate.

#### 4.3.2. Apoptosis Detection by FACS Analysis and Confocal Microscopy

To measure the level of apoptosis in the four tumor cell lines (RAW 264.7, L929, MDA-MB-468 and HeLa) annexin-positive cell membrane staining and propidium iodide nuclear staining were measured in cells treated with DE and P4 dilutions after 6 and/or 24 h by both FACS analysis and confocal microscopy imaging.

To perform the FACS analysis the four cell lines were all seeded in 6-well tissue culture plates, in complete medium, at a density of 400,000 cells/well, except for RAW 264.7 cells that were seeded at a density of 800,000 cells/well. The day after cells were incubated in the presence or absence of different dilutions of DE and P4 (100 and 10 μg/mL) for 6 h or 24 h. At the end of the incubation, the cell media were removed from each wells and cells washed once with phosphate buffer saline (PBS) and treated with a trypsin- Ethylenediaminetetraacetic acid (EDTA) solution in PBS to detach cells still adherent to the plate and then centrifuged at 600× *g* for 5 min at RT. The pelleted cells were then stained using the Annexin V, Fluorescein isothiocyanate (FITC) Apoptosis Detection Kit (Dojindo EU, GmbH) following the manufacturer’s instructions. FACS analysis was then performed on BD FACS Canto II flow cytometer (Becton Dickinson BD Italia spa, Milan, Italy) by FACS DIVA software 6.1.3 version with acquisition criteria of 10,000 events for each tube. The analysis strategy included the following plots and gates for untreated and treated samples: (a) FSC-A vs. SSC-A with a gate for live cells by light scatter properties; (b) FSC-A vs. FSC-H to exclude doublets; and (c) Annexin V FITC-A vs. Propidium Iodide-A (PI-A) to evaluate annexin V-/PI- live cells, annexin V+/PI− early apoptotic cells, annexin V+/PI+ dead cells or apoptotic. The results were expressed as the percentage of apoptotic cells in the live population.

Confocal microscopy images were obtained using a Leica TCS SL confocal microscope equipped with argon/He-Ne laser sources and a HCX PL APO CS 63.0 × 1.40 oil objective (Leica Microsystems, Wetzlar, Germany). For imaging cell apoptosis, the four tumor cell lines (RAW 264.7, L929, MDA-MB-468 and HeLa) were seeded in eight-well Lab-Teck chambered slides (Nalge Nunc Int., Naperville, IL, USA) at 50,000 cells/well, the day after cells were treated with 100 μg/mL P4 for 6 h. Subsequently, live cells were stained using the Annexin V, FITC Apoptosis Detection Kit (Dojindo EU, GmbH) following the manufacturer’s instructions. Images of living cells (2 × digital zoom) were then acquired, in single stacks, both in the phase contrast mode as well as in fluorescence mode by applying a laser energy of 50% to the 488 nm line and acquiring in the emission range of 500–550 nm for the green fluorescence of annexin-positive cells and in the emission range of 600–670 nm for the red fluorescence of propidium iodide-positive cells.

#### 4.3.3. Intracellular Calcium Detection

Experiments were performed in 96-well plates as already described [35]. Briefly, RAW 264.7 cells were plated at a density of 25,000 cells/well, while L929 cells were plated at a density of 10,000 cells/well and allowed to adhere overnight. Cells were then incubated for 45 min at 37 °C with 10 μM Fluo3-AM calcium sensitive dye in complete medium (Life Technologies, Milan, Italy). Subsequently, the wells were washed twice with Hank’s Balanced Salt Solution (HBSS) to remove excess of dye from the medium and afterwards the experiment of intracellular calcium rise detection was performed in extracellular HBSS medium with or without Ca^2+^, the latter also in the presence of 50 μM EDTA calcium chelator. Cells were treated or untreated with 50 or 100 μg/mL of P4 or 50 μg/mL of DE in the presence or absence of Ca^2+^ in the extracellular medium and the plates were immediately read on a Fluostar Optima BMG using 485/520 excitation/emission wavelengths for an interval of 1 h, recording each well fluorescence value every 15 s. Data are the means ± S.D. of two independent experiments performed in triplicate.

#### 4.3.4. Intracellular ROS Detection

Experiments were performed in 96-well plates as already described [36]. Briefly, RAW 264.7 cells were plated at a density of 25,000 cells/well, while L929 cells were plated at a density of 10,000 cells/well and allowed to adhere overnight. Cells were then washed once with HBSS and incubated for 45 min at 37 °C with 10 μM 2′,7′-dichlorodihydrofluorescein diacetate dye in HBSS (Life Technologies, Milan, Italy). Subsequently, cells were washed with HBSS to remove excess of dye from the medium and then stimulation of ROS production was performed in extracellular HBSS medium with or without Ca^2+^, the latter also in the presence of 50 μM EDTA calcium chelator. ROS production was evaluated after 2 h incubation at 37 °C either with 200 μM H_2_O_2_ (positive control), or with 50 or 100 μg/mL of P4 or 50 μg/mL of DE in the presence or absence of Ca^2+^ in the extracellular medium. The plates were finally read on a FLUOstar® Omega multi-mode microplate reader (BMG Labtech, Ortenberg, Germany) using 485/520 excitation/emission wavelengths. Data are the means ± S.D. of two independent experiments performed in which each condition was tested eight times.

### 4.4. Statistical Analysis

Statistical analysis was performed using one-way ANOVA plus Tukey’s post-test (GraphPad Software, Inc., San Diego, CA, USA). *p* values < 0.05 were considered significant.

## Figures and Tables

**Table 1:** The proteins identified from 2D (two-dimensional) gel spots of peaks 4 and 5 by Q Exactive™ nano- electrospray ionization coupled with tandem mass spectrometry (ESI-MS/MS) and SEQUEST search engine. For each protein (isotig) the following details are reported: Score (Sequest), coverage (% of the protein sequence covered by identified peptides), unique peptides (unique peptides in the protein group), peptides (distinct peptides in the protein group).

P4						
Spot	C. R. Database Id	Score	Coverage	Proteins	Unique Peptides	Peptides
**1**	isotig13644	207.90	58.29	1	10	11
**2**	isotig13644	133.15	58.29	1	8	11
**3**	isotig13644	137.50	58.29	1	8	11
**4**	isotig13644	237.20	58.29	1	11	11
**5**	isotig11703	66.86	37.37	1	4	10
**6**	isotig13644	19.68	45.23	1	7	8
**6**	isotig02770	74.19	45.81	2	2	6
**6**	isotig02771	47.81	45.81	2	2	6
**P5**						
**Spot**	**C. R. Database Id**	**Score**	**Coverage**	**Proteins**	**Unique peptides**	**Peptides**
**1**	isotig13644	379.61	58.29	1	12	12
**2**	isotig13644	340.54	58.29	1	10	12
**3**	isotig13644	181.69	58.29	1	11	11
**4**	isotig13644	309.34	58.29	1	12	12

**Table 2:** Identified proteins. For each identified protein total amino acid number, theoretical MW and IP are given. Moreover, the table indicates the accession number, the protein description and the organism in which the proteins displaying the maximum alignment score (sorted by E-value), obtained by the National Center for Biotechnology Information (NCBI) Protein Blast analysis, are identified.

C. R. Database Id	aa	MW (kDa)	IP	Accession Number	Description	Organism	E Value
isotig13644	199	21.9	5.27	WP_142038611.1	DNA-binding response regulator	*Arthrobacter sp.*	5.9
isotig11703	281	31.9	5.76	ABR53885.1	Astrosclerin-1	*Astrosclera willeyana*	7.30 × 10^−61^
isotig02770	203	23.3	4.59	XP_016993171.1	PREDICTED: ubiquitin-conjugating enzyme E2 H	*Drosophila takahashii*	4.4
isotig02771	203	23.3	4.55	XP_016993171.1	PREDICTED: ubiquitin-conjugating enzyme E2 H	*Drosophila takahashii*	4.4

## Supplementary Materials

The following are available online at https://www.mdpi.com/1660-3397/18/8/409/s1, Supplementary File S1. Chondrosin alignment using Phyre2 (see methods). Particularly relevant the high confidence with ryanodine receptor and the short identity with a region of beta chain of defensins.
