# Supplementary material for: Identification, Purification and Molecular Characterization of Chondrosin, a New Protein with Anti-tumoral Activity from the Marine Sponge Chondrosia Reniformis Nardo 1847
Source: Mar Drugs. 2020 Aug 2;18(8):409. doi: 10.3390/md18080409 (PMC7459819; doi:10.3390/md18080409)
Supplement: Supplementary file 1 [file marinedrugs-18-00409-s001.pdf]

Phyre2

Email	mgiovine@unige.it
Description	University
Date	Wed Jun 17 16:51:04 BST 2020
Unique Job ID	7fa6611bbc4ced80

Detailed template information

#	Template	Alignment Coverage	3D Model	Confidence	% i.d.	Template Information
1	c3ilaG_	Alignment	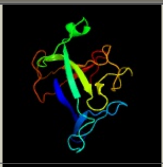	86.0	18	**PDB header:**signaling protein<br>**Chain:** G: **PDB Molecule:**ryanodine receptor 1;<br>**PDBTitle:** crystal structure of rabbit ryanodine receptor 1 n-terminal domain (9-2 205)
2	d2cqea1	Alignment	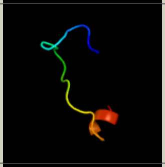	49.4	36	**Fold:**CCCH zinc finger<br>**Superfamily:**CCCH zinc finger<br>**Family:**CCCH zinc finger
3	c1xzzA_	Alignment	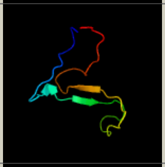	31.2	29	**PDB header:**membrane protein<br>**Chain:** A: **PDB Molecule:**inositol 1,4,5-trisphosphate receptor type 1;<br>**PDBTitle:** crystal structure of the ligand binding suppressor domain of type 12 inositol 1,4,5-trisphosphate receptor
4	c3qr5B_	Alignment	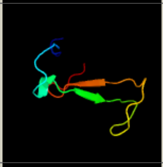	29.4	22	**PDB header:**signaling protein<br>**Chain:** B: **PDB Molecule:**cardiac ca2+ release channel;<br>**PDBTitle:** structure of the first domain of a cardiac ryanodine receptor mutant2 with exon 3 deleted
5	d2cspa1	Alignment	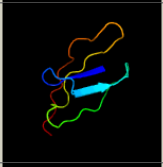	27.1	28	**Fold:**Immunoglobulin-like beta-sandwich<br>**Superfamily:**Fibronectin type III<br>**Family:**Fibronectin type III
6	c4qslC_	Alignment	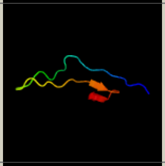	19.9	37	**PDB header:**ligase<br>**Chain:** C: **PDB Molecule:**pyruvate carboxylase;<br>**PDBTitle:** crystal structure of listeria monocytogenes pyruvate carboxylase
7	c4b4rA_	Alignment	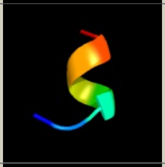	18.2	88	**PDB header:**cell adhesion<br>**Chain:** A: **PDB Molecule:**f18 fimbrial adhesin ac;<br>**PDBTitle:** crystal structure of the lectin domain of f18 fimbrial2 adhesin fedf in complex with blood group b type 13 hexasaccharide
8	c4n7vB_	Alignment	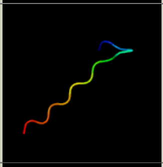	17.9	43	**PDB header:**cell cycle<br>**Chain:** B: **PDB Molecule:**serine/threonine-protein kinase plk4;<br>**PDBTitle:** crystal structure of human plk4 cryptic polo box (cpb) in complex with2 a cep152 n-terminal fragment
9	c4nhyC_	Alignment	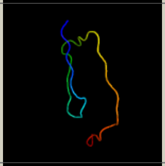	17.7	24	**PDB header:**oxidoreductase/oxidoreductase inhibitor<br>**Chain:** C: **PDB Molecule:**2-oxoglutarate and iron-dependent oxygenase domain-<br>**PDBTitle:** crystal structure of human ogfod1, 2-oxoglutarate and iron-dependent2 oxygenase domain containing 1, in complex with pyridine-2,4-3 dicarboxylic acid (2,4-pdca)
10	c3pe0B_	Alignment	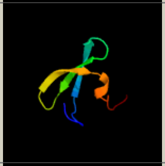	17.2	17	**PDB header:**structural protein<br>**Chain:** B: **PDB Molecule:**plectin;<br>**PDBTitle:** structure of the central region of the plakin domain of plectin
11	d1rutx3	Alignment	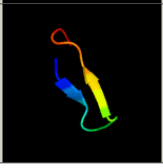	16.7	40	**Fold:**Glucocorticoid receptor-like (DNA-binding domain)<br>**Superfamily:**Glucocorticoid receptor-like (DNA-binding domain)<br>**Family:**LIM domain

12	c2l0zA_	Alignment	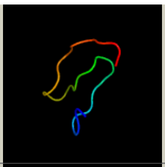	16.7	32	**PDB header:**viral protein<br>**Chain:** A: **PDB Molecule:**glycoprotein g2;<br>**PDBTitle:** solution structure of a zinc-binding domain from the junin virus2 envelope glycoprotein
13	c5y9vA_	Alignment	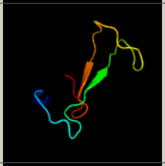	14.9	20	**PDB header:**transport protein<br>**Chain:** A: **PDB Molecule:**ryanodine receptor 1;<br>**PDBTitle:** crystal sturcture of diamondback moth ryanodine receptor n-terminal2 domain
14	c2xoaA_	Alignment	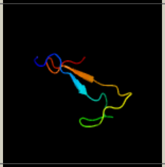	13.9	23	**PDB header:**metal transport<br>**Chain:** A: **PDB Molecule:**ryanodine receptor 1;<br>**PDBTitle:** crystal structure of the n-terminal three domains of the2 skeletal muscle ryanodine receptor (ryr1)
15	c6iuqA_	Alignment	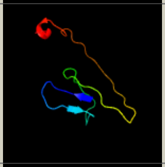	11.5	27	**PDB header:**oxidoreductase<br>**Chain:** A: **PDB Molecule:**prolyl 4-hydroxylase;<br>**PDBTitle:** crystal structure and expression patterns of prolyl 4-hydroxylases2 from phytophthora capsici
16	d1q9ha_	Alignment	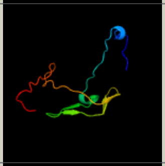	11.3	33	**Fold:**Concanavalin A-like lectins/glucanases<br>**Superfamily:**Concanavalin A-like lectins/glucanases<br>**Family:**Glycosyl hydrolase family 7 catalytic core
17	c4ojpC_	Alignment	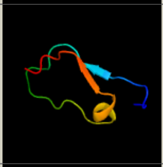	11.2	38	**PDB header:**viral protein<br>**Chain:** C: **PDB Molecule:**tailspike protein;<br>**PDBTitle:** crystal structure of putative tailspike protein (tsp1, orf210) from2 escherichia coli o157:h7 bacteriohage cba120 in complex with maltose
18	c2b68A_	Alignment	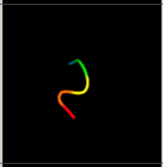	11.0	100	**PDB header:**antimicrobial protein<br>**Chain:** A: **PDB Molecule:**defensin;<br>**PDBTitle:** solution structure of the recombinant crassostrea gigas2 defensin
19	c2rfyB_	Alignment	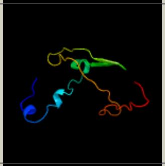	11.0	22	**PDB header:**hydrolase<br>**Chain:** B: **PDB Molecule:**cellulose 1,4-beta-cellobiosidase;<br>**PDBTitle:** crystal structure of cellobiohydrolase from melanocarpus albomyces2 complexed with cellobiose
20	c4zzpB_	Alignment	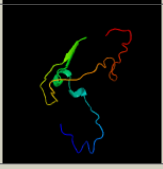	11.0	25	**PDB header:**hydrolase<br>**Chain:** B: **PDB Molecule:**cellulose 1,4-beta-cellobiosidase;<br>**PDBTitle:** dictyostelium purpureum cellobiohydrolase cel7a apo structure
21	d2icya2	Alignment	not modelled	10.8	32	**Fold:**Nucleotide-diphospho-sugar transferases<br>**Superfamily:**Nucleotide-diphospho-sugar transferases<br>**Family:**UDP-glucose pyrophosphorylase
22	c4l4iA_	Alignment	not modelled	9.6	23	**PDB header:**metal transport<br>**Chain:** A: **PDB Molecule:**ryanodine receptor 2;<br>**PDBTitle:** crystal structure of mouse ryanodine receptor isoform 2 (ryr2) 1-5472 disease mutant r420q
23	c5j3tC_	Alignment	not modelled	9.6	67	**PDB header:**hydrolase<br>**Chain:** C: **PDB Molecule:**edc1;<br>**PDBTitle:** crystal structure of s. pombe dcp2:dcp1:edc1 mrna decapping complex
24	c5o60K_	Alignment	not modelled	9.4	30	**PDB header:**ribosome<br>**Chain:** K: **PDB Molecule:**50s ribosomal protein l13;<br>**PDBTitle:** structure of the 50s large ribosomal subunit from mycobacterium2 smegmatis
25	c5n2vC_	Alignment	not modelled	9.0	67	**PDB header:**rna binding protein<br>**Chain:** C: **PDB Molecule:**edc1;<br>**PDBTitle:** changes in conformational equilibria regulate the activity of the dcp22 decapping enzyme
26	c5n2vF_	Alignment	not modelled	9.0	67	**PDB header:**rna binding protein<br>**Chain:** F: **PDB Molecule:**edc1;<br>**PDBTitle:** changes in conformational equilibria regulate the activity of the dcp22 decapping enzyme
27	c2dhkA_	Alignment	not modelled	8.9	32	**PDB header:**immune system<br>**Chain:** A: **PDB Molecule:**tbc1 domain family member 2;<br>**PDBTitle:** solution structure of the ph domain of tbc1 domain family2 member 2 protein from human
28	c5jp4B_	Alignment	not modelled	8.9	67	**PDB header:**peptide binding protein<br>**Chain:** B: **PDB Molecule:**uncharacterized protein c18g6.09c;<br>**PDBTitle:** crystal structure of s. pombe dcp1 in complex with the decapping2 enhancer edc
						**PDB header:**transferase

29	c2lulA_	Alignment	not modelled	8.7	14	**Chain:** A: **PDB Molecule:**tyrosine-protein kinase tec; **PDBTitle:** solution nmr structure of ph domain of tyrosine-protein kinase tec2 from homo sapiens, northeast structural genomics consortium (nesg)3 target hr3504c
30	c1pxqA_	Alignment	not modelled	8.3	83	**PDB header:**antimicrobial protein **Chain:** A: **PDB Molecule:**subtilisin a; **PDBTitle:** structure of subtilisin a
31	c5ergB_	Alignment	not modelled	8.3	15	**PDB header:**transferase **Chain:** B: **PDB Molecule:**trna (adenine(58)-n(1))-methyltransferase catalytic subunit **PDBTitle:** crystal structure of the two-subunit trna m1a58 methyltransferase2 trm6-trm61 in complex with sam
32	c2jopA_	Alignment	not modelled	8.2	46	**PDB header:**immune system **Chain:** A: **PDB Molecule:**t-cell surface glycoprotein cd5; **PDBTitle:** solution structure of the n-terminal extracellular domain of the2 lymphocyte receptor cd5 (cd5 domain 1)
33	c3malA_	Alignment	not modelled	8.1	33	**PDB header:**plant protein **Chain:** A: **PDB Molecule:**stromal cell-derived factor 2-like protein; **PDBTitle:** crystal structure of the sdf2-like protein from arabidopsis thaliana
34	d1x1fa1	Alignment	not modelled	8.1	18	**Fold:**PH domain-like barrel **Superfamily:**PH domain-like **Family:**Pleckstrin-homology domain (PH domain)
35	c6qyyD_	Alignment	not modelled	8.0	50	**PDB header:**virus **Chain:** D: **PDB Molecule:**capsid fiber protein; **PDBTitle:** the crystal structure of head fiber gp8.5 n base in bacteriophage2 phi29
36	c6bj5F_	Alignment	not modelled	7.3	71	**PDB header:**immunosuppressant **Chain:** F: **PDB Molecule:**serine proteinase inhibitor 1; **PDBTitle:** structure of the clinically used myxomaviral serine protease inhibitor2 1 (serp-1)
37	c4m6tA_	Alignment	not modelled	7.2	31	**PDB header:**transcription regulator **Chain:** A: **PDB Molecule:**rna polymerase ii-associated factor 1 homolog, linker, rna **PDBTitle:** structure of human paf1 and leo1 complex
38	c2jnvA_	Alignment	not modelled	7.0	40	**PDB header:**metal transport **Chain:** A: **PDB Molecule:**nifu-like protein 1, chloroplast; **PDBTitle:** solution structure of c-terminal domain of nifu-like2 protein from oryza sativa
39	c2qsdB_	Alignment	not modelled	6.4	45	**PDB header:**structural genomics, unknown function **Chain:** B: **PDB Molecule:**uncharacterized conserved protein; **PDBTitle:** crystal structure of a protein il1583 from idiomarina loihiensis
40	c4mnoA_	Alignment	not modelled	6.0	23	**PDB header:**translation **Chain:** A: **PDB Molecule:**translation initiation factor 1a; **PDBTitle:** crystal structure of aif1a from pyrococcus abyssi
41	c1t2yA_	Alignment	not modelled	5.9	58	**PDB header:**metal binding protein **Chain:** A: **PDB Molecule:**metallothionein; **PDBTitle:** nmr solution structure of the protein part of cu6-2 neurospora crassa mt
42	c2lg1A_	Alignment	not modelled	5.8	19	**PDB header:**metal binding protein **Chain:** A: **PDB Molecule:**a-kinase anchor protein 13; **PDBTitle:** solution structure of the human akap13 ph domain and stabilizing dh2 helix
43	d1xkia_	Alignment	not modelled	5.8	8	**Fold:**Lipocalins **Superfamily:**Lipocalins **Family:**Retinol binding protein-like
44	d1xhja_	Alignment	not modelled	5.8	27	**Fold:**Alpha-lytic protease prodomain-like **Superfamily:**Fe-S cluster assembly (FSCA) domain-like **Family:**NifU C-terminal domain-like
45	c2q4jB_	Alignment	not modelled	5.7	40	**PDB header:**transferase **Chain:** B: **PDB Molecule:**probable utp-glucose-1-phosphate uridylyltransferase 2; **PDBTitle:** ensemble refinement of the protein crystal structure of gene product2 from arabidopsis thaliana at3g03250, a putative udp-glucose3 pyrophosphorylase
46	d1knxa1	Alignment	not modelled	5.7	10	**Fold:**MurF and HprK N-domain-like **Superfamily:**HprK N-terminal domain-like **Family:**HPr kinase/phoshatase HprK N-terminal domain
47	d1k78a2	Alignment	not modelled	5.6	41	**Fold:**DNA/RNA-binding 3-helical bundle **Superfamily:**Homeodomain-like **Family:**Paired domain
48	d1efna_	Alignment	not modelled	5.5	14	**Fold:**SH3-like barrel **Superfamily:**SH3-domain **Family:**SH3-domain
49	c2v6zM_	Alignment	not modelled	5.5	20	**PDB header:**transferase **Chain:** M: **PDB Molecule:**dna polymerase epsilon subunit 2; **PDBTitle:** solution structure of amino-terminal domain of human dna polymerase2 epsilon subunit b
50	d1gpia_	Alignment	not modelled	5.4	38	**Fold:**Concanavalin A-like lectins/glucanases **Superfamily:**Concanavalin A-like lectins/glucanases **Family:**Glycosyl hydrolase family 7 catalytic core
51	c4afzC_	Alignment	not modelled	5.4	11	**PDB header:**hydrolase/de novo protein **Chain:** C: **PDB Molecule:**fynomer; **PDBTitle:** human chymase - fynomer complex
52	d1nxza1	Alignment	not modelled	5.4	19	**Fold:**PUA domain-like **Superfamily:**PUA domain-like **Family:**YggJ N-terminal domain-like
53	c2zkrj_	Alignment	not modelled	5.4	15	**PDB header:**ribosomal protein/rna **Chain:** J: **PDB Molecule:**rna expansion segment es15 part ii; **PDBTitle:** structure of a mammalian ribosomal 60s subunit within an 80s complex2 obtained by docking homology models of the rna and proteins into an3 8.7 a cryo-em map
54	d6paxa2	Alignment	not modelled	5.3	41	**Fold:**DNA/RNA-binding 3-helical bundle **Superfamily:**Homeodomain-like

						**Family:**Paired domain
55	c2yg1A_	Alignment	not modelled	5.2	30	**PDB header:**hydrolase<br>**Chain:** A: **PDB Molecule:**cellulose 1,4-beta-cellobiosidase;<br>**PDBTitle:** apo structure of cellobiohydrolase 1 (cel7a) from heterobasidion2 annosum
56	d1o98a1	Alignment	not modelled	5.2	21	**Fold:**2,3-Bisphosphoglycerate-independent phosphoglycerate mutase, substrate-binding domain<br>**Superfamily:**2,3-Bisphosphoglycerate-independent phosphoglycerate mutase, substrate-binding domain<br>**Family:**2,3-Bisphosphoglycerate-independent phosphoglycerate mutase, substrate-binding domain
57	c2k06A_	Alignment	not modelled	5.2	26	**PDB header:**transcription<br>**Chain:** A: **PDB Molecule:**transcription antitermination protein nusg;<br>**PDBTitle:** solution structure of the aminoterminal domain of e. coli nusg
58	d1ojja_	Alignment	not modelled	5.2	21	**Fold:**Concanavalin A-like lectins/glucanases<br>**Superfamily:**Concanavalin A-like lectins/glucanases<br>**Family:**Glycosyl hydrolase family 7 catalytic core
59	d1h8ac1	Alignment	not modelled	5.1	33	**Fold:**DNA/RNA-binding 3-helical bundle<br>**Superfamily:**Homeodomain-like<br>**Family:**Myb/SANT domain
